# Attenuated lipotoxicity and apoptosis is linked to exogenous and endogenous augmenter of liver regeneration by different pathways

**DOI:** 10.1371/journal.pone.0184282

**Published:** 2017-09-06

**Authors:** Thomas S. Weiss, Madeleine Lupke, Sara Ibrahim, Christa Buechler, Julia Lorenz, Petra Ruemmele, Ute Hofmann, Michael Melter, Rania Dayoub

**Affiliations:** 1 Children’s University Hospital, University of Regensburg, Regensburg, Germany; 2 Center for Liver Cell Research, University of Regensburg Hospital, Regensburg, Germany; 3 Department of Internal Medicine, University of Regensburg Hospital, Regensburg, Germany; 4 Institute of Pathology, University Hospital Erlangen, Friedrich-Alexander University Erlangen-Nuernberg, Erlangen, Germany; 5 Dr. Margarete Fischer-Bosch Institute of Clinical Pharmacology and University of Tübingen, Stuttgart, Germany; 6 Department of Biochemistry and Microbiology, Faculty of Pharmacy, Damascus University, Damascus, Syria; University College London, UNITED KINGDOM

## Abstract

Nonalcoholic fatty liver disease (NAFLD) covers a spectrum from simple steatosis to nonalcoholic steatohepatitis (NASH) and cirrhosis. Free fatty acids (FFA) induce steatosis and lipo-toxicity and correlate with severity of NAFLD. In this study we aimed to investigate the role of exogenous and endogenous ALR (augmenter of liver regeneration) for FFA induced ER (endoplasmatic reticulum) -stress and lipoapoptosis. Primary human hepatocytes or hepatoma cells either treated with recombinant human ALR (rhALR, 15kDa) or expressing short form ALR (sfALR, 15kDa) were incubated with palmitic acid (PA) and analyzed for lipo-toxicity, -apoptosis, activation of ER-stress response pathways, triacylglycerides (TAG), mRNA and protein expression of lipid metabolizing genes. Both, exogenous rhALR and cytosolic sfALR reduced PA induced caspase 3 activity and Bax protein expression and therefore lipotoxicity. Endogenous sfALR but not rhALR treatment lowered TAG levels, diminished activation of ER-stress mediators C-Jun N-terminal kinase (JNK), X-box binding protein-1 (XBP1) and proapoptotic transcription factor C/EBP-homologous protein (CHOP), and reduced death receptor 5 protein expression. Cellular ALR exerts its lipid lowering and anti-apoptotic actions by enhancing FABP1, which binds toxic FFA, increasing mitochondrial β-oxidation by elevating the mitochondrial FFA transporter CPT1α, and decreasing ELOVL6, which delivers toxic FFA metabolites. We found reduced hepatic mRNA levels of ALR in a high fat diet mouse model, and of ALR and FOXA2, a transcription factor inducing ALR expression, in human steatotic as well as NASH liver samples, which may explain increased lipid deposition and reduced β-oxidation in NASH patients. Present study shows that exogenous and endogenous ALR reduce PA induced lipoapoptosis. Furthermore, cytosolic sfALR changes mRNA and protein expression of genes regulating lipid metabolism, reduces ER-stress finally impeding progression of NASH.

## Introduction

Nonalcoholic fatty liver disease (NAFLD) has emerged as the most common cause of liver disease in the developed countries in both adults and children [1–3]. NAFLD encompasses a spectrum of hepatic pathologies, ranging from simple steatosis to nonalcoholic steatohepatitis (NASH), liver fibrosis, cirrhosis and hepatocellular carcinoma (HCC) [1, 4]. Obesity, insulin resistance, and increased serum levels of saturated free fatty acids (FFAs) are strongly associated with progression of NAFLD [5]. Despite the high prevalence of NAFLD and its potential for serious complications, a deeper understanding of the underlying mechanisms that determine the progression to liver damage is necessary to develop effective therapies for NAFLD/NASH.

ALR, augmenter of liver regeneration, (encoded by *Gfer* [growth factor Erv1 homolog of *Saccharomyces cerevisiae*]), a member of the ALR/Erv1 protein family, was shown to augment the process of liver regeneration, and therefore, is denoted as a co-mitogen [6]. Hepatic ALR, originally identified in regenerating livers of rats [6] is mainly expressed in hepatocytes and cholangiocytes [7]. ALR functions as a FAD-linked sulfhydryl oxidase catalyzing disulfide bond formation [8, 9], as a cytochrome c reductase [10] and in concert with the redox-regulated receptor Mia40/TOM, ALR forms a disulfide relay system mediating the import of proteins into the mitochondrial inner-membrane system (IMS) [11, 12]. Furthermore, ALR was described to have a functional role as a protective and anti-apoptotic cell survival factor [13–16].

A recent report provides a novel scenario in which liver specific depletion of ALR (ALR-L-KO) was shown to be linked to accelerated development of steatohepatitis and HCC [17]. ALR-L-KO mice at age of two weeks showed reduced mitochondrial respiratory function, increased oxidative stress and had extensive steatosis and apoptosis shown by increased Bax expression and caspase 3 activation. Furthermore, ALR depletion resulted in decreased expression of genes involved in lipid metabolism such as carnitine palmitoyl transferase 1 (CPT1α), and ATP synthesis such as ATP synthase subunit ATP5G1. Enhanced lipid accumulation in ALR-L-KO mice was probably a consequence of impaired mitochondrial fatty acid β-oxidation, consistent with reduced CPT1α levels, while lipogenesis was unchanged [17].

Several studies have implicated the involvement of endoplasmatic reticulum (ER) -stress in lipoapoptosis and lipotoxicity [18–20]. The hepatic ER is responsible for protein maturation, lipid synthesis, detoxification and cellular calcium storage. Chronic activation of ER-stress was shown to be linked to metabolic disease e.g. NAFLD and insulin resistance [21] and plays a role in development of diabetes in obesity [22]. There are three trans-membrane-ER proteins known to sense and transduce ER-stress response: protein kinase RNA-like ER kinase (PERK), inositol-requiring protein-1α (IRE-1α) and activating transcription factor 6 (ATF6). Their downstream mediators serve as ER-stress markers. ATF6 and PERK activation results in expression of the proapoptotic transcription factor C/EBP-homologous protein (CHOP), while IRE-1α activation leads to C-Jun N-terminal kinase (JNK) activation, and also creates a spliced form of X-box binding protein-1 (XBP1) mRNA, a transcription factor that promotes degradation of misfolded ER proteins [19].

In this study we analyzed the changes on hepatic lipotoxicity and lipoapoptosis after re-expression (endogenous) of 15 kDa, short form ALR (sfALR) and treatment (exogenous) with 15 kDa recombinant human ALR (rhALR). We investigated activation of ER-stress response pathways after FFA treatment and analyzed triacylglyceride (TAG) levels as well as expression of genes involved in lipid metabolism. Furthermore we analyzed ALR and FOXA2 (HNF3β), a transcriptional regulator of ALR expression, in liver samples of a high fat diet mouse model and patients with hepatosteatosis or NASH.

## Materials and methods

### Human liver samples

Human liver tissues were histologically examined for patients without NAFLD (n = 17), patients with simple liver steatosis (n = 27) and patients with NASH (n = 29) as described earlier [23] (for tissue characteristics see supplementary S1 Table) and mRNA expression of ALR, FOXA2 and YWHAZ (primer sequences are listed in supplementary S2 Table) were analyzed. Experimental procedures were performed according to the guidelines of the charitable state controlled foundation HTCR (Human Tissue and Cell Research, Regensburg, Germany), with the written informed patient's consent. The study and the consent form were approved by the local ethical committee of the University of Regensburg (ethics statement 12-101-0048, University of Regensburg, Germany). All experiments involving human tissues and cells have been carried out in accordance to *The Code of Ethics of the World Medical Association* (Declaration of Helsinki).

### HFD mouse model

Mouse liver samples were obtained from mice as described in detail elsewhere [24, 25]. Briefly, fourteen week old male C57BL/6 mice were kept on a high-fat diet (HFD) or standard chow (SD) for 14 weeks. The six mice on a high fat diet had a body weight of 39.3 (32.5–41.3) g, which was significantly higher compared to the five mice on a standard diet with 25.8 (23.9–27.5) g. Rising concentrations of CO_2_ were used to produce loss of consciousness and was followed by cervical dislocation.

All animal experiments were approved by the institutional committee of Animal Care and Use, University of Regensburg (54–2532.1-30/13; Regierung der Oberpfalz, Germany) and conducted in accordance with the German federal law regarding the protection of animals and 'Guide for the Care and Use of Laboratory Animals' (National Institutes of Health publication 8th Edition, 2011).

### RNA isolation, cDNA-synthesis and quantification of mRNA expression by real-time PCR

Total RNA was isolated using RNeasy Mini Kit (Qiagen, Hilden, Germany). One μg of total RNA was reverse-transcribed using the Reverse-Transcription System (Promega, Madison, WI, USA). Transcript levels of ACC, ALR, ATP5G1, CPT1α, ELOVL6, FABP1, FASN, FOXA2, HPRT, PPARα, SCD1, SREBP1c, TFAM, and YWHAZ (primer sequences are listed in supplementary S2 Table) were quantified using real-time PCR technology (Light-Cycler, Roche, Penzberg, Germany). PCR reaction products were verified by sequence analysis and PCR analysis was performed in triplicates.

### Western blot analysis

Total protein fractions (20 μg per lane) were separated by 14% SDS-PAGE under reducing conditions using 50 mM DTT. Proteins were transferred onto polyvenylidene fluoride membranes, incubated with specific antibodies for ALR, Bax, CHOP, CPT1α, CVα, DR5, eIF2-α, phospho-eIF2-α, FABP1, GAPDH, HSP70, JNK, phospho-JNK, SCD1 (see supplementary material) and developed with ECL reactions (Pierce, Rockford, IL, USA).

### Nile Red staining

Cells were grown on glass coverslips. After treatment, intracellular neutral lipid was stained using Nile Red (2 μg/ml) for 5 min at room temperature. Cells were then fixed with 4% paraformaldehyde for 15 min at room temperature. Afterwards, cells were washed with PBS and mounted in Prolong Antifade (Invitrogen, Camarillo, CA) for subsequent microscopy.

### Immunocytochemistry for ALR and FOXA2 localization

Cells grown on glass coverslips were stained using rabbit anti-ALR (1:50), mouse anti-Golgi 97 (1:100), mouse anti-PDI (1:100), MitoTracker^®^ Deep Red FM or goat anti-FOXA2 (1:500). Fluorescent signals were visualized by an Alexa Fluor 488 dye-conjugated goat anti-rabbit (IgG) antibody (Invitrogen), FITC dye-conjugated donkey anti-goat (IgG) antibody (Santa Cruz Biotechnology) or Alexa-Fluor 633 dye-conjugated goat anti-mouse (IgG) antibody (life technologies).

### LDH release measurement

The cell membrane integrity was determined by measuring the release of cytoplasmic lactate dehydrogenase (LDH) using the LDH Tox7-1 in vitro Assay Kit (Sigma-Aldrich, St. Louis, MO) according to the manufacturer’s instructions.

### Caspase 3/7 activity measurement

Following treatments, cells were subjected to Caspase 3/7 activity measurement with Caspase-Glo^®^ 3/7 Assay (Promega, Madison, USA) according to the manufacturer’s instructions.

### Preparation of mitochondria

Mitochondria were isolated from primary human hepatocytes, HepG2 and HepG2-sfALR cells using the Mitochondria Isolation Kit for Cultured Cells (Life Technologies, Darmstadt, Germany) following the manufacturer’s instructions.

### ATP assay

After FFA treatment for 24 h, cells were lysed and cellular ATP content was measured using Luminescent ATP detection assay (Abcam, Germany) following manufacturer’s instructions.

### Statistical analysis

All data are presented as mean values +/- standard deviation. Statistical analysis for non parametric data was done by two-tailed Mann-Whitney U Test, Student’s t-test for paired samples, and value of p < 0.05 was regarded as significant (SPSS Statistics 21.0 program, IBM, Leibiz Rechenzentrum, München, Germany).

More detailed information on materials and methods are described in the supplementary S1 File.

## Results

### Expression and subcellular localization of endogenous ALR

**Fig 1:**
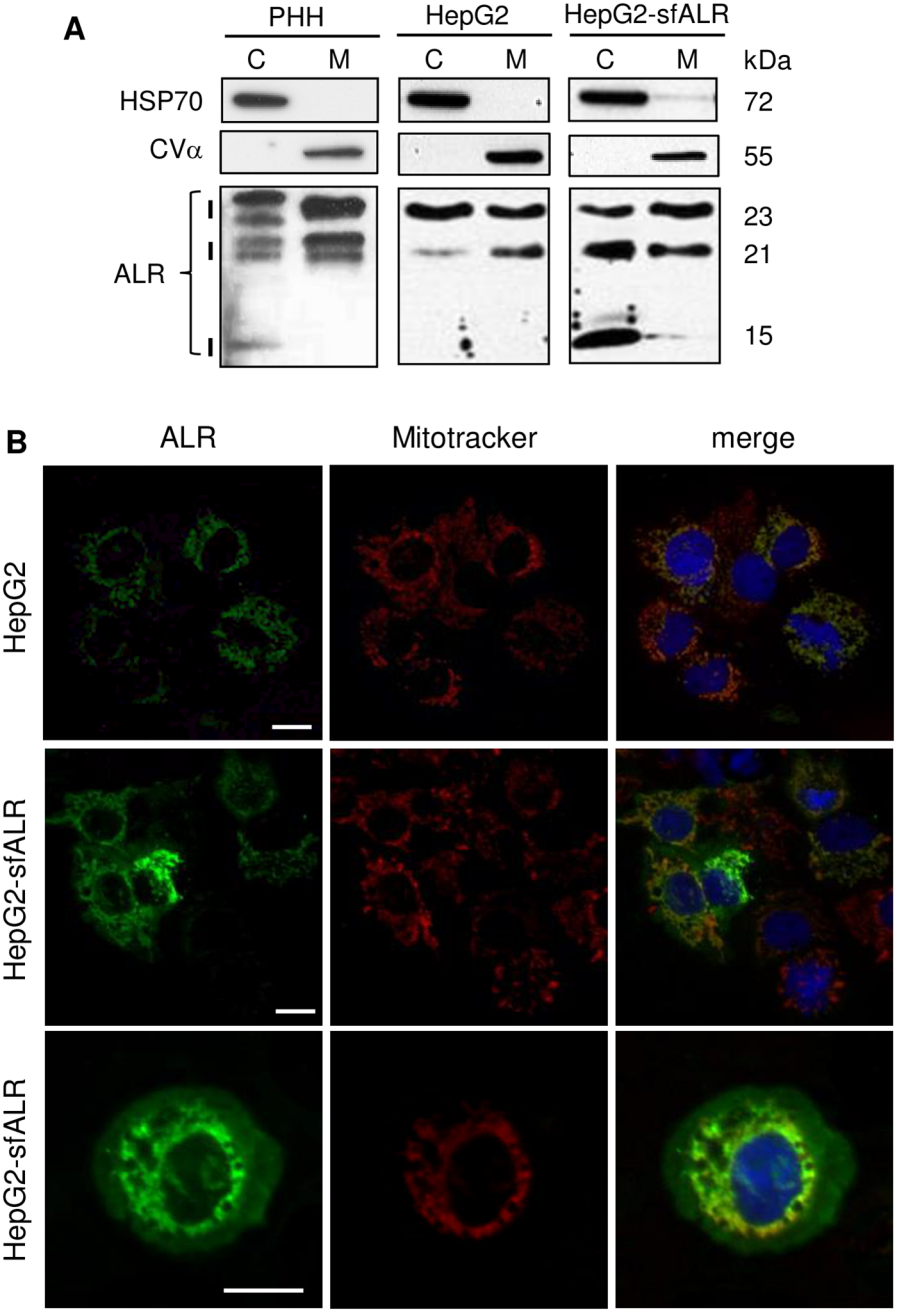
ALR is expressed in different isoforms and subcellular localizations (1). (A) PHH, HepG2 and HepG2 cells stably expressing sfALR (HepG2-sfALR) were separated into mitochondrial, M, and cytosolic, C, fractions followed by western blot analysis of ALR isoform expression. Specificity of subcellular fractions is demonstrated by expression of specific markers for cytosol, HSP70, and mitochondria, CVα. ALR isoforms of 23 kDa and 21 kDa are found in cytosolic as well as mitochondrial fractions, whereas 15 kDa ALR is only detected in cytosolic fractions of PHH with a faint and in HepG2-sfALR cells with an intense signal. (B) Immunocytochemistry of ALR expression in HepG2 and HepG2-sfALR cells. Cells were immuno-stained for ALR (green) and mitochondria (red, Mitotracker) visualizing co-localization by yellow overlap in the merge image. ALR is expressed in the cytosol and in mitochondria with a more intense immune signal in the cytosol of HepG2-sfALR cells.

**Fig 2:**
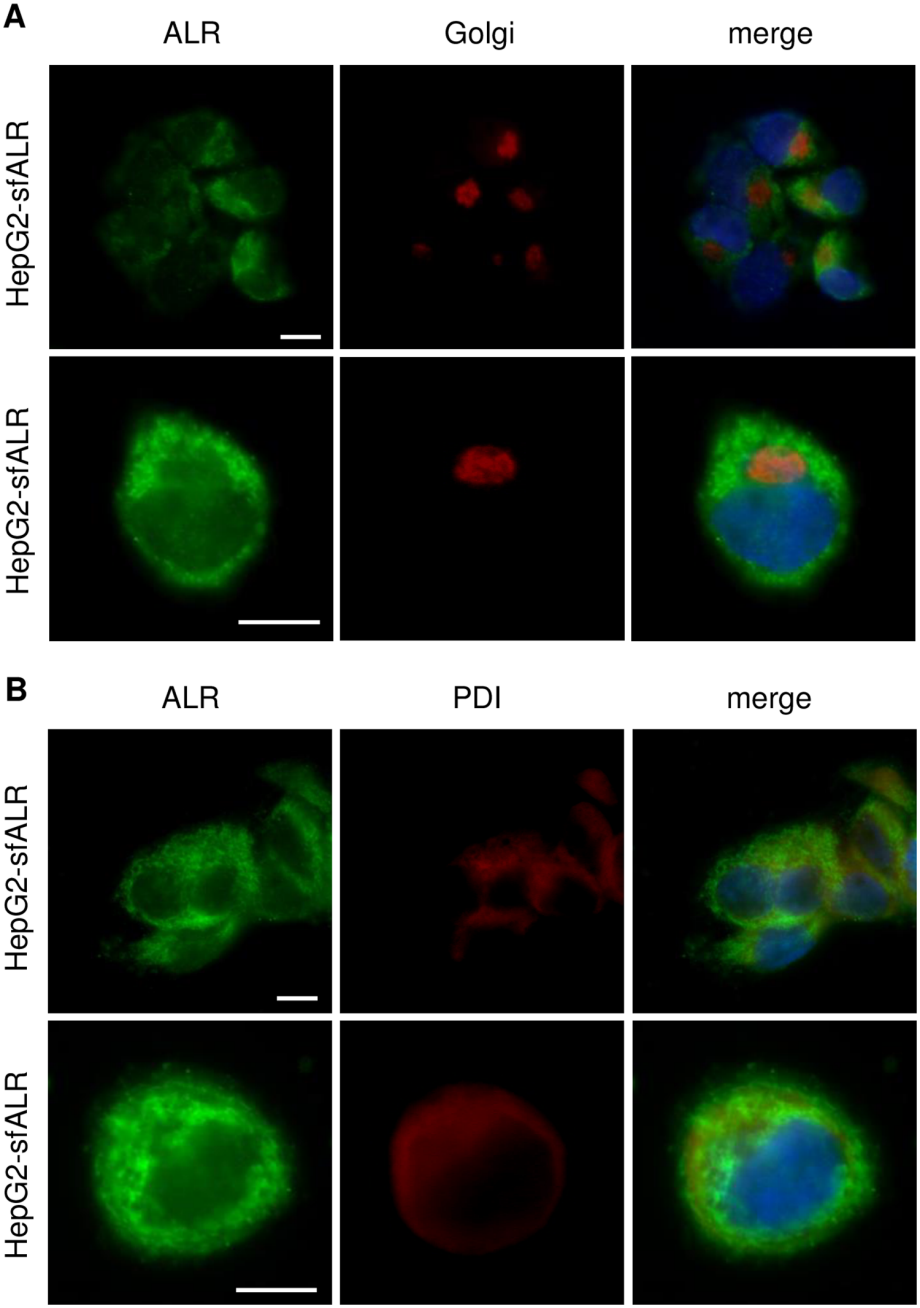
ALR is expressed in different isoforms and subcellular localizations (2). Immunocytochemistry of ALR expression in HepG2 and HepG2-sfALR cells. Cells were immuno-stained for ALR (green) and no co-localization (yellow overlap in the merge image) was observed with (a) Golgi apparatus (red, Golgi-marker) nor with (B) ER (red, protein disulfide isomerase).

Native ALR protein appears on western blots under reducing conditions as three bands of 15 kDa, 21 kDa and 23 kDa [26, 27]. All three isoforms are expressed in liver tissue, but their cellular localization is less clear. Subcellular separation of cytosolic and mitochondrial fractions of PHH revealed expression of both, 21 and 23 kDa ALR in mitochondria and cytosol, the latter with an additional weak expression of 15 kDa ALR (Fig 1A). Hepatoma cells express two ALR isoforms, 21 kDa and 23 kDa (long form, lfALR; S1A Fig). Subcellular analysis of HepG2 cells showed both forms in mitochondria and cytosol. HepG2 cells stably expressing 15 kDa ALR (short form, sfALR), HepG2-sfALR, demonstrated sfALR only in the cytosolic fraction (Fig 1A). Specificity of anti-ALR antibody was verified by si-RNA transfection experiments silencing expression of all ALR isoforms in HepG2 and Huh7 cells (S1C Fig). Immunostaining of ALR in HepG2 and HepG2-sfALR cells confirms cellular localization of ALR. Immunosignals for HepG2 are in mitochondria and faint staining in the cytosol, and for HepG2-sfALR cells in mitochondria and intense staining in the cytosol (Fig 1B). Localization is demonstrated by co-immunostaining with mitochondrial markers. Furthermore absence of co-localization of ALR and Golgi as well as ER marker expression excludes localization of ALR in Golgi-apparatus and ER (Figs 1 and 2).

### ALR reduces lipotoxicity and lipoapoptosis

**Fig 3:**
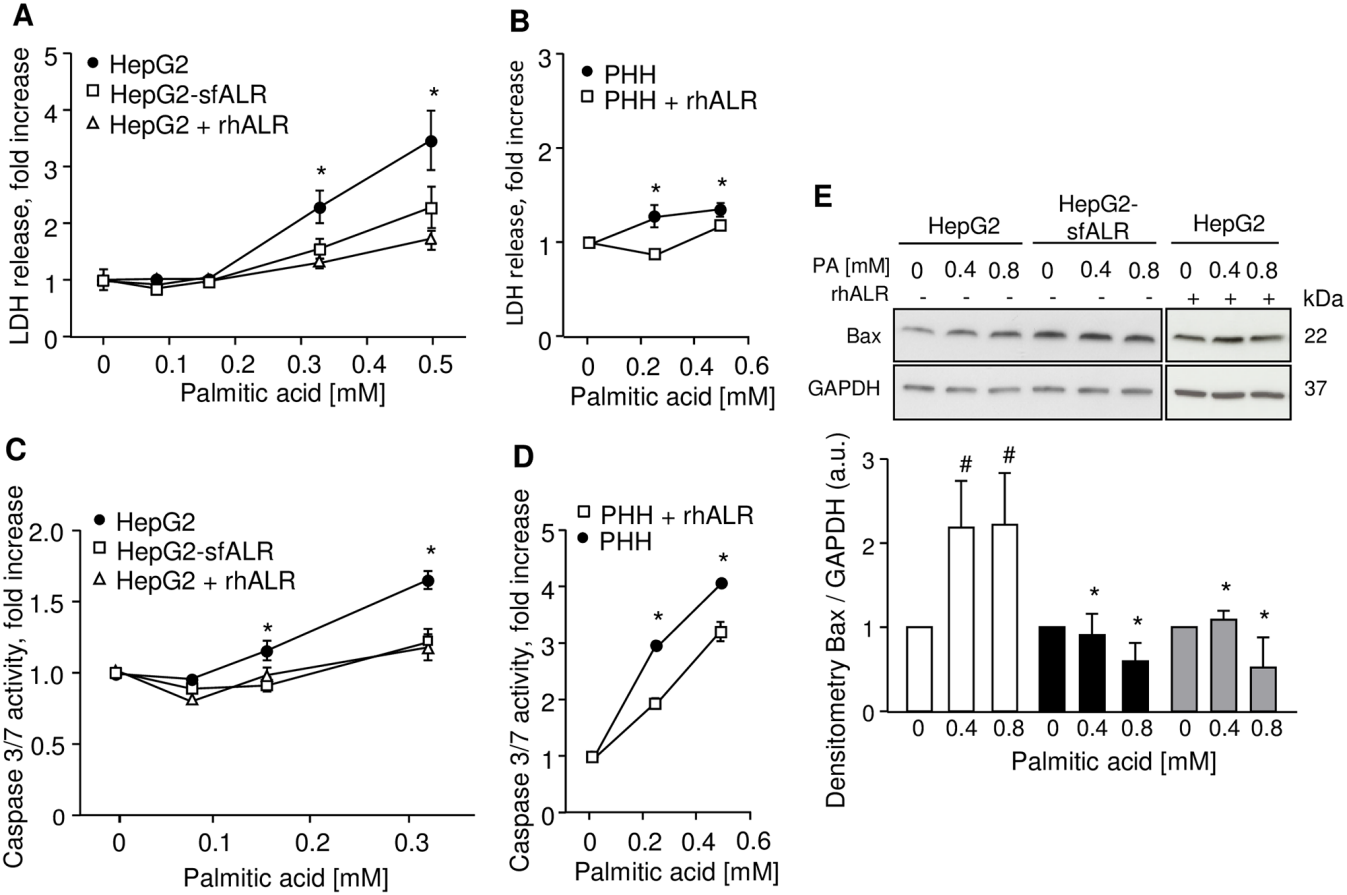
Treatment of hepatic cells with PA results in lipotoxicity and lipoapoptosis, which is ameliorated by ALR. Cultured cells were incubated for 24 hours with indicated concentrations of palmitic acid or vehicle (0 mM PA). (A), (B) Lipotoxicity, analyzed by LDH release into supernatant, is reduced in HepG2-sfALR (0.33 mM, 0.5 mM) and HepG2 (0.33 mM, 0.5 mM) as well as PHH (0.25 mM, 0.5 mM) pretreated with 100 nM rhALR compared to HepG2 and PHH without rhALR treatment. (C), (D) Lipoapoptosis, analyzed by cellular caspase 3/7 activity, is reduced in HepG2-sfALR (0.16 mM, 0.33 mM) and HepG2 (0.16 mM, 0.33 mM) as well as PHH (0.25 mM, 0.5 mM) pretreated with 100 nM rhALR compared to HepG2 and PHH without rhALR treatment. (n = 3; * p < 0.05 differs from HepG2-sfALR, HepG2 + rhALR or PHH + rhALR). (E) Bax protein expression is reduced in HepG2-sfALR and HepG2 treated with 100 nM rhALR compared to HepG2 without rhALR treatment. Cells were incubated with either 0 mM (vehicle), 0.4 mM or 0.8 mM PA for 8 hours. Activation of Bax was demonstrated by western-blot analysis (n = 3; * p < 0.05 differs from corresponding HepG2, # p < 0.05 differs from 0 mM PA).

Treatment of HepG2 and Huh7 cells with increasing concentrations of palmitic acid (PA) causes lipo-toxicity and apoptosis as seen by enhanced LDH release (Fig 3A and S2A Fig) and caspase 3/7 activity (Fig 3C and S2B Fig), respectively, which is reduced by expression of sfALR in HepG2-sfALR or Huh7-sfALR cells. In addition, application of recombinant human ALR (rhALR) to hepatoma cells or PHH diminished PA induced lipo-toxicity and apoptosis (Fig 3A–3D). Furthermore, induction of Bax protein expression after PA treatment is blunted for HepG2 cells stably expressing sfALR or treated with rhALR indicating an anti-lipoapoptotic effect of ALR (Fig 3E).

### Cytosolic sfALR expression alleviates PA induced ER-stress

**Fig 4:**
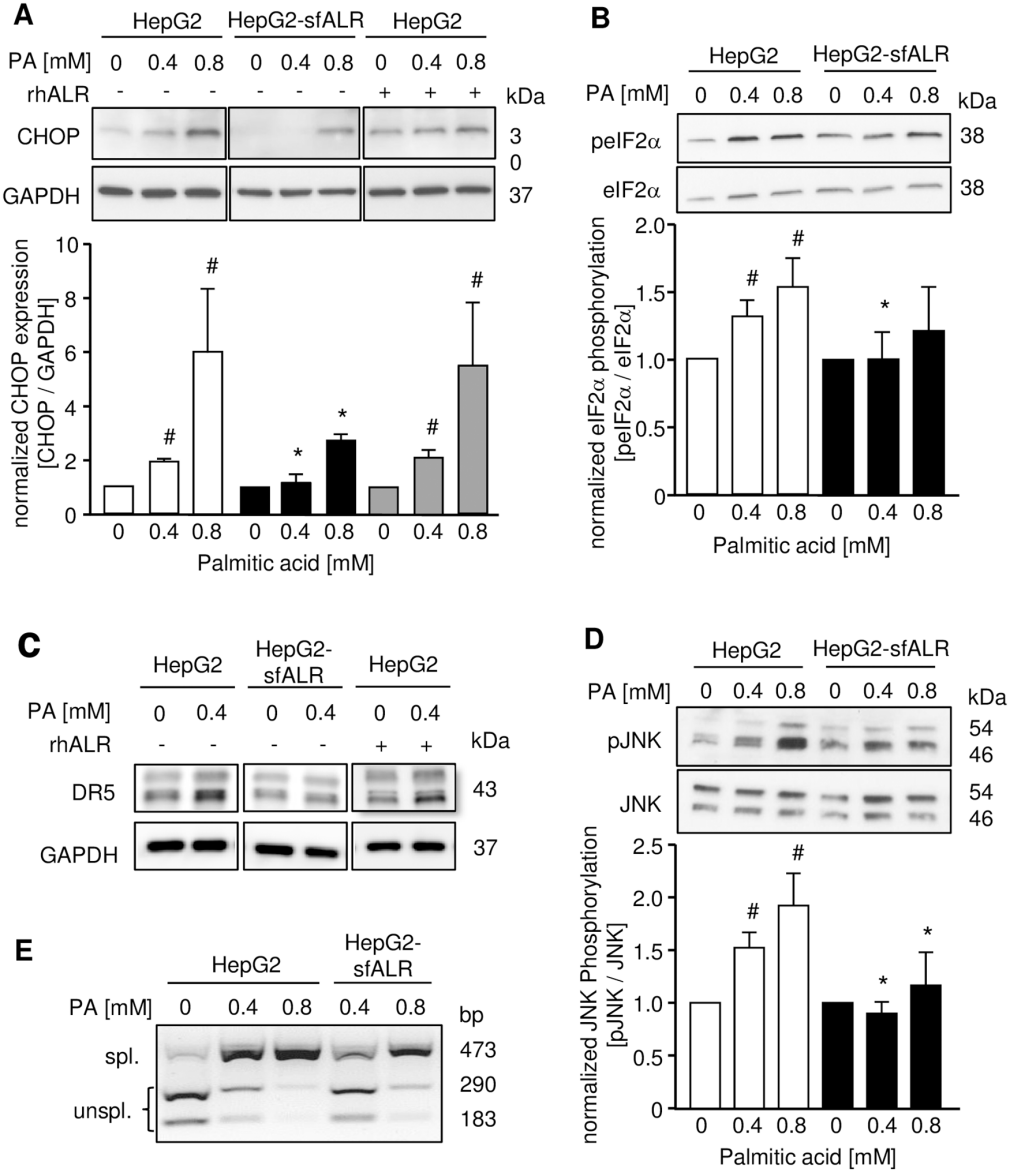
Endogenous sfALR reduces PA-induced ER-stress and death receptor expression. Cultured cells were incubated for 8 hours with indicated concentrations of palmitic acid or vehicle (0 mM PA). (A) Expression of CHOP was analyzed by western blotting lysates of HepG2-sfALR and HepG2 cells treated without or with 100 nM rhALR followed by densitometric analysis. (B) Activation of elF2α by phosphorylation was analyzed by western blotting lysates of HepG2 and HepG2-sfALR cells followed by densitometric analysis. (n = 3; * p < 0.05 differs from corresponding HepG2, # p < 0.05 differs from 0 mM PA). (C) Expression of death receptor 5 (DR5) after 24 hours incubation with PA was analyzed by western blotting. (D) Activation of JNK by phosphorylation was analyzed by western blotting lysates of HepG2 and HepG2-sfALR cells followed by densitometric analysis (n = 3; * p < 0.05 differs from corresponding HepG2, # p < 0.05 differs from 0 mM PA). (E) Activation of XBP1 in HepG2 and HepG2-sfALR cells was analyzed by amplifying XBP1 cDNA by PCR following incubation with P*st*I. Spliced form of XBP1 indicating activation results in a 473 bp amplification product, whereas unspliced form of XBP1 shows digested 290 bp and 183 bp amplification products (one out of three experiments is shown).

**Fig 5:**
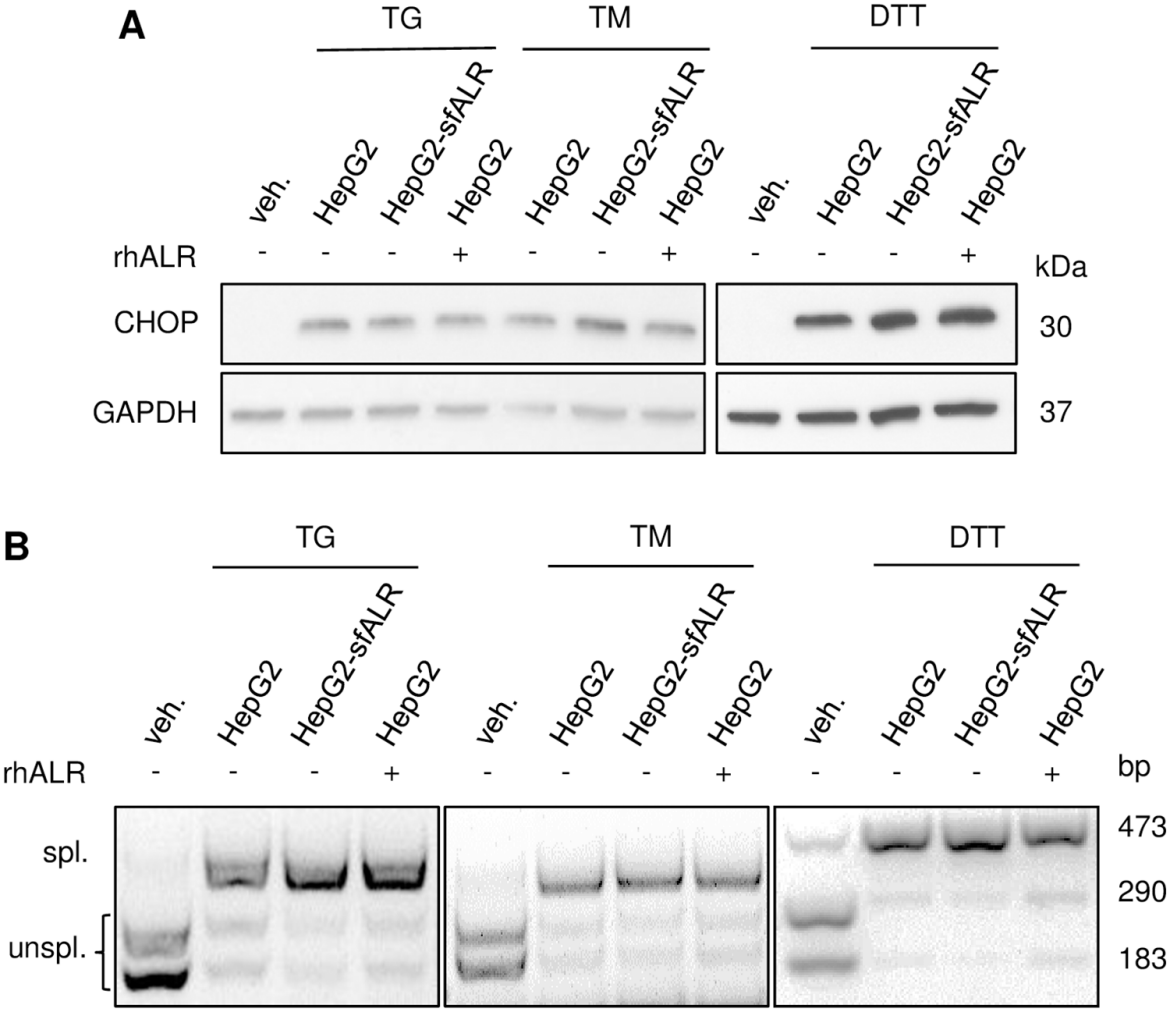
ER-stress induced by Thapsigargin, Tunicamycin or DTT is not attenuated by ALR. Cultured cells were incubated either for 6 hours with 0.5 μM Thapsigargin, for 16 hours with 10 μg/ml Tunicamycin or 2 mM DTT. (A) Activation of CHOP was analyzed by western blotting of HepG2-sfALR and HepG2 cells treated without or with 100 nM rhALR. (B) Activation of XBP1 in HepG2-sfALR and HepG2 cells treated without or with 100 nM rhALR was analyzed by amplifying XBP1 cDNA by PCR followed by incubation with P*st*I. Spliced form indicating activation results in a 473 bp amplification product, whereas unspliced form of XBP1 shows digested 290 bp and 183 bp amplification products (one out of three experiments is shown).

Treatment of hepatoma cells with PA increases lipoapoptosis involving ER-stress pathways [18, 19], which is seen by enhanced expression of proapoptotic transcription factor CHOP (Fig 4A and S3A Fig). In contrast, HepG2 or Huh7 cells expressing sfALR revealed significant less CHOP expression at 0.4 mM and 0.8 mM PA treatment and hence reduced expression of death receptor 5 [19] after PA treatment compared to HepG2 cells (Fig 4C). Interestingly, treatment of HepG2 or Huh7 cells with rhALR did not lower PA induced CHOP expression (Fig 4A and S3A Fig) pointing to different molecular mechanisms how ALR mediates an anti-apoptotic effect which is dependent on its cellular localization. Furthermore, we examined activation of ER-stress response transducer PERK by analyzing activation of eukaryotic initiation factor 2 (elF2α), which is upstream of CHOP and activated by PERK [19]. ALR expression attenuated phosphorylation of elF2α upon PA treatment in HepG2 and Huh7 cells (Fig 4B and S3B Fig). Next, we explored ER-stress response transduction by IRE-1, which is characterized by JNK activation and XBP1 splicing [19]. Activating phosphorylation of JNK by PA (0.4 mM, 0.8 mM) was reduced (Fig 4D and S3C Fig) as well as PA induced XBP1 splicing was inhibited by sfALR expression compared to wild type cells (Fig 4E). XBP1 splicing indicating activation of IRE-1 endonuclease activity was not altered by rhALR upon PA treatment in hepatoma cells and PHH (S3D Fig), which underlines that sole cytosolic sfALR reduces PA induced ER-stress pathways. Additionally, we analyzed if ALR lowers ER-stress response transduction induced by thapsigargin (TG), tunicamycin (TM) or dithiothreitol (DTT) representing classical cell biological ER-stress inducers [19]. Neither sfALR expression nor rhALR treatment reduced or inhibited TG, TM or DTT-induced CHOP expression as well as XBP1 splicing, respectively (Fig 5A and 5B and S4A and S4B Fig). In summary, cytosolic sfALR expression, but not exogenously applied rhALR blocks activation of ER-stress response pathways induced particularly by PA, a saturated cytotoxic FFA.

### Cytosolic sfALR expression reduces cellular steatosis, alters expression of genes with a role in lipid metabolism and increases ATP levels

**Fig 6:**
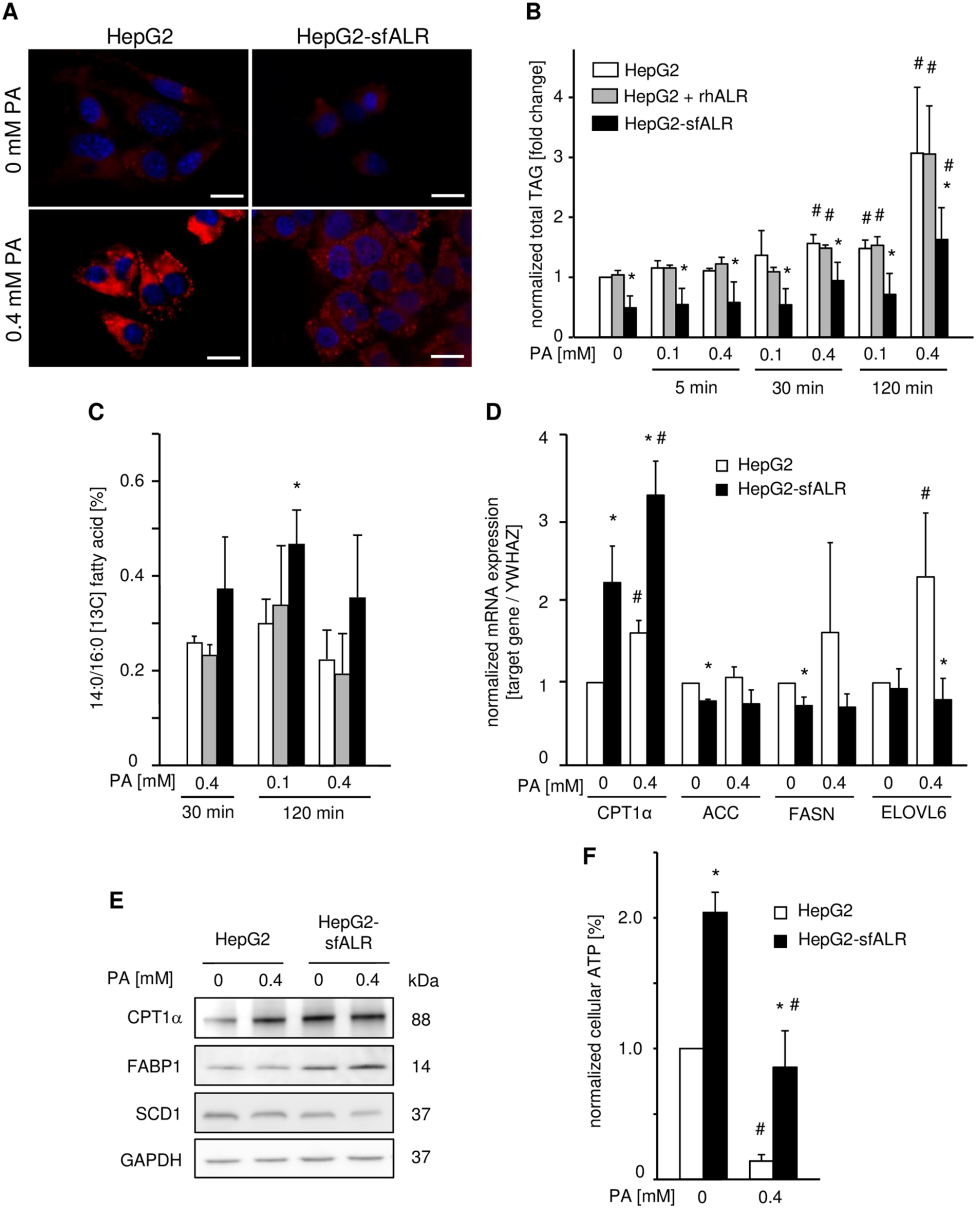
Endoenous ALR results in less cellular lipid content, alters lipid metabolism associated genes and enhances ATP levels. (A) Cultured cells were incubated for 24 hours with indicated concentrations of palmitic acid or vehicle. Cellular lipid content of HepG2 and HepG2-sfALR cells was analyzed by staining lipid droplets using Nile Red following fluorescence microscopy (one out of three experiments is shown; bar = 20 μm). (B) Total TAG levels were analyzed in HepG2-sfALR and HepG2 cells (without or with 100 nM rhALR) incubated with PA for the indicated time. Alteration of total TAG content is shown corresponding to HepG2 cells (n = 3; * p < 0.05 differs from HepG2; # p < 0.05 differs from 0 mM PA). (C) Cultured cells (HepG2-sfALR and HepG2 without or with 100 nM rhALR) were incubated with [13C] PA for the indicated time and analyzed for content of [13C]-labelled fatty acids. Ratio of 14:0/16:0 in percent is shown (n = 3). (D) HepG2-sfALR and HepG2 cells were incubated for 24 hours with palmitic acid or vehicle and analyzed for lipid metabolism related gene expression (CPT1α, ACC, FASN, ELOVL6 mRNA) performing qRT-PCR. YWHAZ mRNA expression was determined for normalization (n = 3). (E) Cell homogenates from cells decribed in (D) were further analyzed by western blotting (one out of three experiments is shown). (F) Cellular ATP content was determined in HepG2 and HepG2-sfALR cells treated for 24 hours with palmitic acid or vehicle (n = 3; * p < 0.05 differs from HepG2; # p < 0.05 differs from corresponding 0 mM PA).

Treatment of HepG2 and Huh7 cells with 0.4 mM PA resulted in enhanced lipid droplet formation, which is diminished in cells expressing sfALR (Fig 6A). In addition, formation of triacylglycerides (TAGs), the main constituents of lipid droplets, was reduced in HepG2-sfALR compared to HepG2 cells after treatment with [13C] PA, but not in HepG2 cells treated with rhALR (Fig 6B). The observed differences in TAG levels were not due to altered import of PA into the cell, because cellular uptake rate of PA was almost unchanged for up to two hours of [13C] PA treatment (Table 1). Analysis of lipid metabolism related genes revealed enhanced CPT1α (carnitine palmitoyltransferase Iα) mRNA expression in HepG2-sfALR cells (Fig 6D), which is responsible for fatty acid transport into mitochondria fueling β-oxidation. Expression of ACC (acetyl-CoA carboxylyse A), which catalyzes synthesis of malonyl-CoA, an inhibitor of CPT1α, and FASN (fatty acid synthase) were unchanged in sfALR cells after PA treatment (Fig 6D). Western blotting confirmed enhanced CPT1α expression in HepG2-sfALR cells compared to HepG2 cells (Fig 6E) and in addition, an increase in FABP1 (fatty acid binding protein 1) was observed, a cellular fatty acid transporter (Fig 6E). Expression of sfALR might results in enhanced activity of mitochondrial β-oxidation due to increased CPT1α and FABP1 expression, and hence increased 14:0 fatty acid content (Fig 6C), which is substantiated by elevated ATP levels (Fig 6F and S6C Fig). Expression of ELOVL6 (elongation of long-chain fatty acids family member 6) regulating FA chain elongation from C16 to C18, was reduced in PA treated HepG2-sfALR (Fig 6D). Reduced ELOVL6 expression in sfALR expressing cells might be responsible for diminished levels of TAG 52:2, which is the most prevalent TAG form (S5 Fig). Notably, TAG 48:1 and TAG 50:2 levels (with 16:1 as main constituent) are increased for sfALR expressing cells (S5 Fig) and this suggestion is supported by a tendency to higher 16:1 / 16:0 ratios in these cells (Table 1). A conclusion would be a higher activity of SCD1 (stearoyl-CoA desaturase 1), incorporating double bands to C16:0 or C18:0 fatty acids, but we found slightly reduced SCD1 mRNA (S6B Fig) and almost no change in SCD1 protein expression (Fig 6E). Viability of mitochondria was not affected by PA treatment and sfALR expression as shown by mitochondrial gene expression (S6A Fig).

**Table 1:** Uptake rate of [13C] palmitic acid and percentage of 13C-labelled fatty acids to [13C] palmitic acid in Huh7 cells at indicated time points.

	13C-PA		5 min			30 min			120 min	
	[mM]	Huh7	Huh7 + ALR	Huh7-sfALR	Huh7	Huh7 + ALR	Huh7-sfALR	Huh7	Huh7 + ALR	Huh7-sfALR
total uptake ^a^	0.1	1.85 ± 0.19	1.83 ± 0.53	2.29 ± 0.78	7.49 ± 0.82	6.11 ± 0.84	6.41 ± 1.72	22.15 ± 9.17	19.56 ± 4.48	22.51 ± 6.78
	0.4	5.41 ± 0.53	4.85 ± 1.15	7.59 ± 0.81^b^^,^^c^	20.80 ± 7.61	18.80 ± 2.88	23.22 ± 4.15	66.65 ± 10.61	54.45 ± 20.01	79.14 ± 12.88
18:0/16:0 [%]	0.1	n.d.	n.d.	n.d.	1.25 ± 0.18	1.38 ± 0.88	1.12 ± 0.23	1.07 ± 0.44	1.27 ± 0.23	0.91 ± 0.31
	0.4	1.6 ± 0.22	1.59 ± 0.43	1.02 ± 0.46	1.52 ± 0.47	1.53 ± 0.52	1.22 ± 0.18	1.33 ± 0.28	2.39 ± 1.32	1.47 ± 0.38
18:1/16:0 [%]	0.1	n.d.	n.d.	n.d.	n.d.	n.d.	n.d.	1.75 ± 0.35	1.3 ± 0.44	0.85 ± 0.62
	0.4	1.42 ± 0.39	1.34 ± 0.48	0.99 ± 0.51	0.94 ± 0.02	0.99 ± 0.42	0.69 ± 0.16	1.28 ± 0.53	0.97 ± 0.67	1.08 ± 0.12
16:1/16:0 [%]	0.1	7.49 ± 0.34	10.45 ± 1.02	14.99 ± 10.03	11.14 ± 4.85	10.41 ± 5.96	20.17 ± 15.1	15.99 ± 10.74	15.28 ± 9.77	25.26 ± 16.75
	0.4	4.78 ± 1.72	5.82 ± 3.62	9.22 ± 6.66	5.71 ± 2.66	6.03 ± 3.02	10.98 ± 8.19	7.78 ± 3.45	7.91 ± 4.83	13.6 ± 9.64

^a^ relative uptake rates per million cells normalized to internal standard

^b^ p < 0.05 different from Huh7

^c^ p < 0.05 different from Huh7 + ALR

n.d. = not detected

### Reduced ALR and FOXA2/HNF3β expression in steatosis and NASH/NAFLD

**Fig 7:**
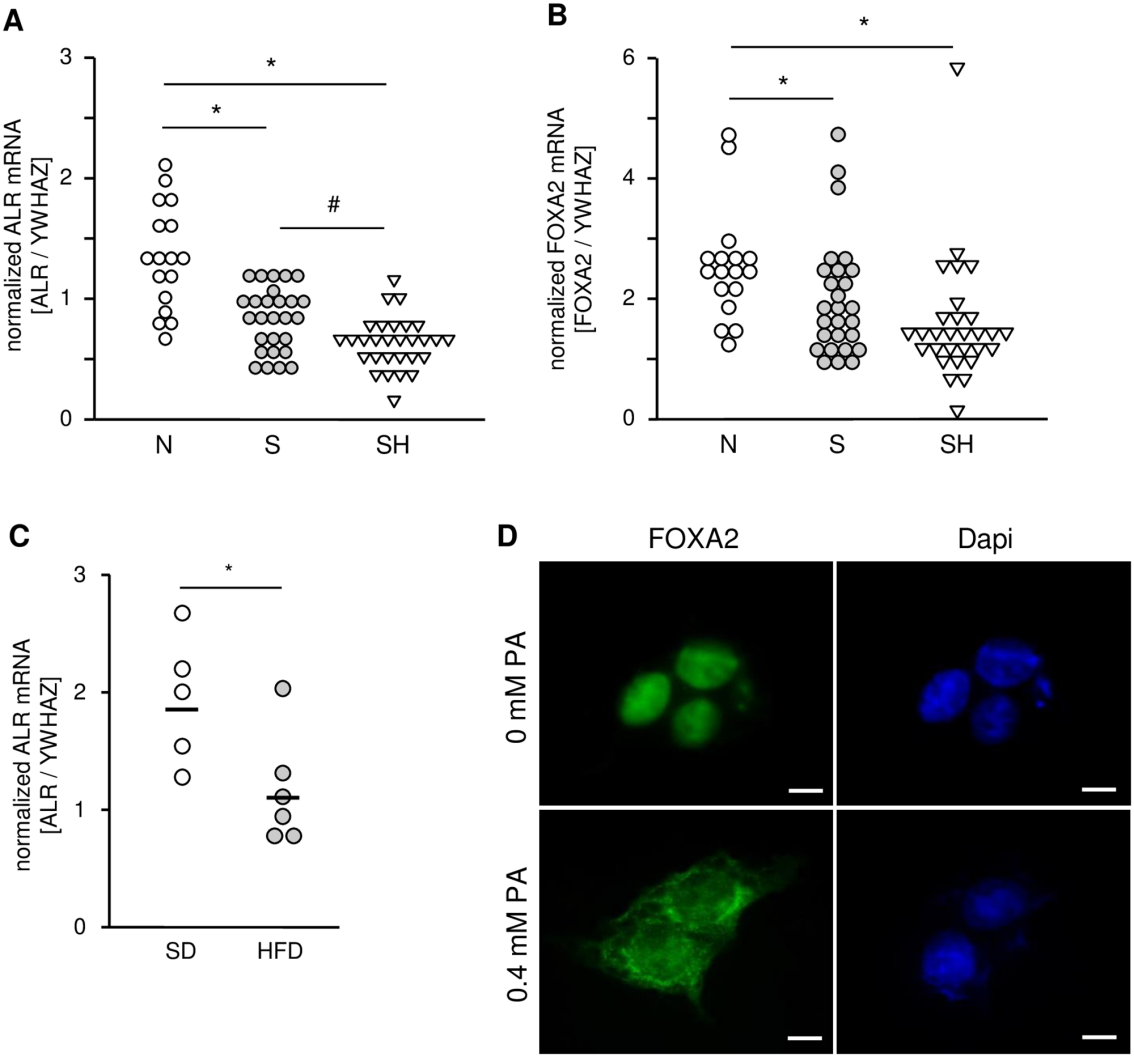
Reduced ALR and FOXA2/HNF3β expression in liver tissue of patients with NAFLD. Expression of (A) ALR mRNA and (B) FOXA2/HNF3β mRNA was analyzed by qRT-PCR in liver tissue samples from patients with hepatic steatosis (n = 27), NASH (n = 29) and normal liver tissue (n = 17). (C) ALR mRNA expression was analyzed by qRT-PCR in liver tissue samples from mice fed a standard diet (SD, lean, n = 5) or high fat diet (HFD, n = 6) for 14 weeks. YWHAZ mRNA expression was determined for normalization (* p < 0.05 differs significantly). (D) HepG2 cells were incubated for 24 hours with 0 mM (vehicle) or 0.4 mM PA and FOXA2/HNF3β translocation from nucleus into cytosol was visualized by immunostaining of FOXA2/HNF3β (one out of three experiments is shown; bar = 10 μm).

Aforementioned results show that diminishing cellular lipid concentration by sfALR expression point to a potential relationship between fatty liver disease and ALR expression. Therefore we analyzed ALR mRNA expression in human liver samples and found significantly reduced ALR mRNA levels in patients with steatosis (0.83 ± 0.26, n = 27) and NASH (0.64 ± 0.22, n = 29) compared to normal liver tissue (1.31 ± 0.48, n = 17) (Fig 7A). Reduction of ALR mRNA expression did not correlate with steatosis grade (S7A Fig). Furthermore, we found reduced ALR mRNA expression in liver samples from mice fed a high fat diet (1.16 ± 0,18, n = 6) compared to standard diet (1.94 ± 0.22, n = 5) (Fig 7C). Liver tissue from patients with NASH showed even lower ALR expression compared to steatotic liver (Fig 7A), but demonstrated no correlation of ALR mRNA to NASH activity score with mild (NASH score 5–6) or severe NASH (NASH score 7–8) (S7B Fig).

We have previously shown that ALR expression is regulated by FOXA2 (HNF3β) [28] and therefore we analyzed FOXA2 mRNA expression in human liver samples with steatosis or NASH. We found a significant reduction of FOXA2 in steatosis (1.98 ± 0.19) and NASH (1.56 ± 0.19) compared to normal liver (2.52 ± 0.22) (Fig 7B), which in part correlates with ALR expression in the same tissue samples (S7C Fig). Nuclear localization and proposed FOXA2 activity, is reduced in HepG2 (Fig 7D) or Huh7 (S7D Fig) cells after treatment with PA demonstrated by its translocation from the nucleus into cytosol [29, 30]. ALR expression is reduced in steatotic and NASH liver tissue which is presumably mediated by a diminished expression and nuclear localization of FOXA2.

## Discussion

Here we report about the ability of sfALR to reduce lipotoxicity and lipoapoptosis upon palmitic acid treatment. This beneficial effect is dependent on the localization of sfALR and based on different molecular mechanisms. While exogenous ALR activates signaling cascades to diminish lipoapoptosis, in addition, cytosolic sfALR ameliorates ER-stress upon palmitic acid treatment and reduces cellular TAG levels while altering lipid metabolism, which results in enhanced beta oxidation and ATP synthesis. Analyzing a cohort of human liver samples we found reduced ALR and FOXA2 expression in steatosis and NASH patients.

ALR protein, as reported previously, is expressed under reducing conditions in three isoforms with molecular sizes of 23, 21 and 15 kDa [26, 27], of which the latter shows a weak expression in human and is not detected in mouse liver tissue (see S1B Fig) [15, 31]. Using human liver cells we found isoforms of 23 and 21 kDa in mitochondria and cytosol, which is in agreement with a reported cytosolic expression of both isoforms [31] or the 23 kDa isoform [32] while analysis of mitochondria in these studies are missing. Furthermore, mitochondrial ALR expression is explained by translocation of lfALR from the cytosol into the mitochondrial intermembrane space due to an intrinsic disordered domain in its N-terminal sequence [33]. Mitochondrial lfALR acts as a disulfide relay system coupled to the oxidoreductase Mia40 facilitating mitochondrial protein import [12]. We have shown in this study that sfALR, lacking the targeting sequence for mitochondria, occurs in PHH as well as in normal human liver tissue and after re-expression in hepatoma cells exclusively in the cytosol. This is in line with reports finding ALR in the cytosol, but also in nucleus as well as released into the extracellular space [6, 34].

Exogenous ALR is found in serum of rats with regenerating livers [31] and in patients with liver diseases [35], as well as to be released by hepatocytes *in vitro* [6]. Furthermore, exogenous rhALR, (corresponds to sfALR) is able to activate EGF receptor (EGF-R) signaling cascades by stimulating tyrosine-phosphorylation of the EGF-R [36] and subsequent activation of Erk1/2-MAPK or PI3K/Akt dependent pathway [36, 37]. Phosphorylation of Akt has been shown to play an essential role in regulating Bcl-2 and Bax expression, and activation of Akt by rhALR results in reduced Bax levels as demonstrated in postischemic rat kidneys [38]. It was reported earlier, that rhALR administration reduces caspase 3 and Bax expression in regenerating rat livers, which was explained by increased expression of anti-apoptotic, soluble clusterin interacting with Bax protein [31]. Furthermore, rhALR lowers apoptotic cell number in neuroblastoma cells treated with H_2_O_2_ due to a diminished intrinsic apoptotic pathway. Interestingly, we found that in steatotic hepatocytes treatment with rhALR does not alter ER-stress apoptosis protein CHOP and death receptor 5 expression, which is regulated by CHOP [19]. Similarly Fas-receptor expression was unchanged in postischemic kidneys after rhALR treatment [38]. Therefore it is likely that exogenous rhALR exerts its anti-apoptotic action by reducing the intrinsic pathway via PI3K/Akt activation and therefore reduced Bax expression, while no impact of rhALR on ER-stress response and extrinsic pathways of apoptosis were observed.

Silencing of ALR gene diminishes expression of all isoforms of ALR leading to increased pro-apoptotic gene expression [15] and deletion of ALR gene in mice results in development of steatohepatitis as well as hepatocellular carcinoma accompanied by ROS generation, mitochondrial damage, and hepatocytes death [17]. It is not clear yet which isoform of ALR contributes to the described cellular alterations [39]. Overexpression of mitochondrial lfALR (23kDa) was shown to increase resistance of hepatoma cells to radiation induced oxidative stress and apoptosis by reducing mitochondrial swelling, loss of ATP production and generation of ROS by enhanced GPx activity [14]. Here in this study we analyzed the role of cytosolic sfALR on fatty acid induced cellular stress and lipoapoptosis. PA exerts its lipotoxic and lipoapoptotic effects by inducing ER-stress and therefore activating ER membrane sensors of the unfolded protein response such as IRE-1α and PERK [19, 40]. Expression of endogenous sfALR, in contrast to exogenous rhALR, diminishes PA induced ER-stress shown by reduced activation of transduction pathways and consecutively CHOP and Bax expression (S8 Fig). We found no direct impact of sfALR on UPR transduction pathways, since perturbation of ER homeostasis by different stimuli such as tunicamycin (inhibits N-linked protein glycosylation), thapsigargin (disrupts calcium stores through inhibition of the sarcoplasmic/endoplasmic reticulum Ca^2+^ ATPase) or dithiothreitol (impairs disulfide bond formation) was not affected by sfALR expression. Therefore it is likely that endogenous sfALR acts upstream of ER processing by changing FFA metabolism.

Endogenous sfALR increases expression of FABP1, which binds long chain fatty acids (LCFA such as PA) and LCFA-CoAs taken over from membranes and therefore minimizes toxic cellular levels of PA and PA-CoA [41]. Furthermore, FABP1 facilitates substrates for both mitochondria and peroxisomes, but enhanced CPT1α expression, as shown for sfALR cells, might favor mitochondrial β-oxidation. Increased mitochondrial β-oxidation attenuates oxidative stress by reducing cellular FFA and alternative oxidation pathways in peroxisomes and microsomes, which results in reduced release of ROS [42, 43]. FABP1 also delivers LCFAs and LCFA-CoAs to the ER for further triacylglyceride synthesis [44], but increased β-oxidation could abate the supply of PA-CoA and therefore decreases TAG synthesis and lipid droplet size (S8 Fig). Recently it was reported that ELOVL6 is involved in NASH related pathogenesis including oxidative stress, inflammatory damage and liver fibrosis [45]. While ELOVL6 expression had only little impact on hepatic lipid content, deficiency in ELOVL6 reduced ROS, liver fibrosis and activation of the inflammasome after PA treatment. In addition, the authors provided evidence that ELOVL6 mediated metabolites, rather than SCD1 mediated desaturation, are mainly responsible for hepatic lipotoxicity [45]. Therefore we hypothesize that reduction of ELOVL6 through sfALR might have similar effects related to NAFLD. In conclusion, endogenous sfALR expression diminishes lipotoxicity by decreasing ELOVL6 mediated toxic metabolites, reducing toxic PA and PA-CoA levels through binding to enhanced FABP1 and therefore together with enhanced CPT1α increases mitochondrial β-oxidation. Reduced levels of PA result in less ER-stress and lower TAG synthesis. Expression of these genes is regulated by various transcription factors among them PPARα for FABP1 and CPT1α [42, 45, 46], and SREBP1c for ELOVL6, SCD1 and CPT1α [42, 46, 47]. There are very few reports describing molecular mechanisms of how sfALR modifies gene or protein expression. sfALR was shown to be localized in the nucleus, binding to JAB1 (c-Jun activating domain-binding protein1), a coactivator of transcription factor AP-1, and thereby increases AP1-activity [27, 48]. Furthermore, sfALR can interact with MIF (macrophage migration inhibitory factor) [49] or thioredoxin [50] regulating AP-1 or NFκ-B activity. Based on these data it is not resolved how endogenous sfALR regulates expression of lipid metabolizing genes, since PPARα and SREBP1c expression were unchanged in sfALR cells (S5C Fig) and therefore further studies are necessary.

In human liver samples expression of ELOVL6 and SCD1 is enhanced in patients with NASH and correlates with inflammation status in hepatic tissue [45, 47]. FABP1 expression is found to be reduced in NASH patients, and therefore, capacity of FFA binding is lowered subsequently worsening lipotoxicity [44]. In this study we showed reduced mRNA expression of ALR, and its transcription factor FOXA2 (HNF3β) [28], in hepatic steatosis and NASH. Furthermore, Nrf2 another ALR regulating transcription factor [51], was reported to have a pivotal role in development of NAFLD, since Nrf2 attenuates liver steatosis by reducing oxidative stress and inhibiting lipid deposition [46]. Therefore it is likely that under the regulation of FOXA2 and Nrf2 diminished endogenous ALR levels in hepatic steatosis result in altered expression of lipid metabolizing genes such as ELOVL6, SCD1, CPT1α as well as FABP1. This may lead to reduced mitochondrial β-oxidation, increased lipid deposition and oxidative stress with enhanced ER-stress and lipoapoptosis finally aggravating the progression of NASH.

## Supporting information

S1 Fig Expression of ALR isoforms in hepatoma cell lines and human, mouse and rat liver tissue. (A) Hepatoma cell lines were transfected with empty vector (pcDNA3.1) or expression plasmid for short form ALR (sfALR, 15 kDa). Positive cell clones stably expressing sfALR (besides 21 and 23 kDa ALR), were selected using G-418 for 2 weeks. (B) Liver tissue homogenates (20 μg) from different species were analyzed for ALR expression performing western blot. Human liver tissue reveals strong bands of 23 and 21 kDa and a weak staining at 15 kDa. Mouse and rat liver show a double band at slightly lower molecular sizes of 22 and 20 kDa, with an additional very faint 15 kDa band in rat liver tissue. (C) HepG2, Huh-7 and Huh-7-sfALR cells were maintained in culture and treated with ALR—siRNA (100 nM) for indicated time points. After 24 and 48 hours ALR expression was analyzed by western blot and demonstrated strong reduction of all ALR isoforms in HepG2, Huh-7 and Huh-7-sfALR cells (one out of three experiments).
(TIF)

S2 Fig ALR ameliorates PA-induced lipotoxicity and lipoapoptosis in hepatic cells. Cultured cells were incubated for 24 hours with indicated concentrations of palmitic acid or vehicle. (A) Lipotoxicity, analyzed by LDH release into supernatant, is reduced in Huh7-sfALR (0.33 mM, 0.5 mM) and Huh7 (0.33 mM, 0.5 mM) pretreated with 100 nM rhALR compared to Huh7 without rhALR treatment. (B) Lipo-apoptosis, analyzed by cellular caspase 3/7 activity, is reduced in Huh7-sfALR (0.16 mM, 0.33 mM) and Huh7 (0.16 mM, 0.33 mM) pretreated with 100 nM rhALR compared to Huh7 without rhALR treatment. (n = 3; * p < 0.05 differs from Huh7-sfALR and Huh7 + rhALR).
(TIF)

S3 Fig Endogenous sfALR diminishes PA-induced apoptosis mediated by ER-Stress. Cultured cells were incubated for 8 hours with indicated concentrations of palmitic acid or vehicle. (A) Expression of CHOP was analyzed by western blotting of Huh7-sfALR and Huh7 cells pretreated without or with 100 nM rhALR followed by densitometric analysis. (B) Activation of elF2α by phosphorylation was analyzed by western blotting of Huh7 and Huh7-sfALR cells followed by densitometric analysis. (n = 3; * p < 0.05 differs from corresponding Huh7, # p < 0.05 differs from 0 mM PA). (C) Activation of JNK by phosphorylation was analyzed by western blotting of Huh7 and Huh7-sfALR cells followed by densitometric analysis. (n = 3; * p < 0.05 differs from corresponding Huh7, # p < 0.05 differs from 0 mM PA). (D) Activation of XBP1 in PHH treated without or with 100 nM rhALR was analyzed by amplifying XBP1 cDNA by PCR following incubation with PstI. Spliced form of XBP1 indicating activation results in a 473 bp amplification product, whereas unspliced form of XBP1 shows digested 290 bp and 183 bp amplification products (one out of three experiments is shown).
(TIF)

S4 Fig ER-stress of PHH induced by Thapsigargin or Tunicamycin is not attenuated by ALR. PHH were incubated either for 6 hours with 0.5 μM Thapsigargin or for 16 hours with 10 μg/ml Tunicamycin. (A) Activation of CHOP was analyzed by western blotting of PHH pretreated without or with 100 nM rhALR. (B) Activation of XBP1 in PHH treated without or with 100 nM rhALR was analyzed by amplifying XBP1 cDNA by PCR following incubation with PstI. Spliced form indicating activation results in a 473 bp amplification product, whereas unspliced form of XBP1 shows digested 290 bp and 183 bp amplification products (one out of three experiments is shown).
(TIF)

S5 Fig Endogenous ALR expression reduces cellular TAG content. Cultured cells were incubated for 24 hours with indicated concentrations of palmitic acid or vehicle. The most abundant individual TAG (48:0, 48:1, 50:1, 50:2, 52:2 and 54:3) levels were analyzed in HepG2-sfALR, HepG2 cells treated without or with 100 nM rhALR. Data were normalized to HepG2 cells treated with 0 mM PA (n = 3; * p < 0.05 differs from HepG2).
(TIF)

S6 Fig Endogenous ALR expression alters lipid metabolism associated gene expression, does not alter mitochondrial gene expression and increases ATP synthesis. Cultured cells were incubated for 24 hours with indicated concentrations of palmitic acid or vehicle. (A) HepG2-sfALR and HepG2 cells were analyzed for ATP5g1 and TFAM mRNA expression performing qRT-PCR. Atp5g1 encodes a subunit of mitochondrial ATP synthase and TFAM (mTFA) encodes mitochondrial transcription factor A, a key activator of mitochondrial transcription and genome replication. HPRT mRNA expression was determined for normalization (n = 3). (B) HepG2-sfALR and HepG2 cells were incubated for 24 hours with palmitic acid or vehicle and analyzed for lipid metabolism related gene expression (PPARα, SREBP1c, FABP1, SCD1 mRNA) performing qRT-PCR. YWHAZ mRNA expression was determined for normalization. (n = 3; * p < 0.05 differs from HepG2; # p < 0.05 differs from 0 mM PA). (C) Cellular ATP content was determined in Huh7 and Huh7-sfALR cells treated for 24 hours with palmitic acid or vehicle (n = 3; * p < 0.05 differs from Huh7; # p < 0.05 differs from corresponding 0 mM PA). ATP content is almost twice as high in sfALR expressing cells as in control cells.
(TIF)

S7 Fig Reduced ALR and FOXA2/HNF3β expression in steatotic and NASH liver tissue. Expression of ALR mRNA was analyzed by qRT-PCR in liver tissue samples from patients with hepatic steatosis or NASH and is plotted in correlation to (A) steatosis grade (<5% = 1.31 ± 0.48; 5–33% = 0.80 ± 0.32; 34–66% = 0.74 ± 0.26; 67–100% = 0.65 ± 0.19) and (B) NASH activity score (normal, N = 1.31 ± 0.48; 1–2 = 0.90 ± 0.24; 3–4 = 0.52 ± 0.06; 5–6 = 0.62 ± 0.21; 7–8 = 0.66 ± 0.23; * p < 0,05 differs significantly. (C) A correlation plot of ALR and FOXA2/HNF3β mRNA expression is shown with best fit lines (Spearman correlation) for NASH (SH, dash line, r^2^ = 0.168), hepatic steatosis (S, dotted line, r^2^ = 0.223) and normal (NO, straight line, r^2^ = 0.235) liver tissue. YWHAZ mRNA expression was determined for normalization. (D) Huh7 cells were incubated for 24 hours with vehicle or 0.4 mM PA and FOXA2/HNF3β translocation from nucleus into cytosol was visualized by immunostaining of FOXA2/HNF3β. (one out of three experiments is shown; bar = 10 μm).
(TIF)

S8 Fig Schematic illustration and summary of the study. 1) FFA such as PA are taken up by the cell and subsequently converted into fatty acyl-CoA. Fatty acyl-CoA (e.g. PA-CoA) are activated forms of FA that can be either oxidized in mitochondria or utilized in the ER as substrates for the synthesis of phospholipids, cholesterol esters, and TAGs. High levels of PA-CoA results in oversaturated lipid intermediates and TAGs, which is responsible for ER-stress and apoptosis [19, 42]. 2) Exogenous applied recombinant human ALR (rhALR) binds to its receptor leading to PI-3K (Akt/PKB) activation [37], which was shown to be responsible for lower expression of pro-apoptotic bax protein and therefore reduced apoptosis. Exogenously applied rhALR could diminish PA induced lipoapoptosis, but had no impact on PA induced activation of ER-stress response pathways. 3) Expression of endogenous ALR leads to altered expression of genes regulating lipid metabolism and subsequently to reduced ER-stress and lipoapoptosis. Cells expressing sfALR show high expression of FABP1 facilitating substrates for both mitochondria and peroxisomes, but enhanced CPT1α expression might favor mitochondrial β-oxidation accompanied by increased ATP levels. Increased mitochondrial β-oxidation attenuates oxidative stress by reducing alternative oxidation pathways in peroxisomes and microsomes, which results in reduced release of ROS. FABP1 also delivers FA-CoAs to the ER for further TAG synthesis, but increased β-oxidation could abate the supply of PA-CoA and therefore decreases TAG synthesis and lipid droplet size. In addition ALR lowers ELOVL6 expression and therefore less ELOVL6 mediated metabolites, which are responsible for hepatic lipotoxicity [45]. Reduced levels of PA, as a consequence of sfALR expression, result in lower TAG synthesis and in less ER-stress shown by reduced activation of ER membrane sensors of the unfolded protein response such as IRE-1α and PERK, and consecutively CHOP and Bax expression. 4) ALR expression is regulated by its transcription factor FOXA2 (HNF3β) [28] and Nrf2 [51] and it is likely that under their regulation diminished endogenous ALR levels in hepatic steatosis result in altered expression of lipid metabolizing genes such as ELOVL6, CPT1α as well as FABP1. ALR, augmenter of liver regeneration; CHOP, C/EBP-homologous protein; CPT1α, carnitine palmitoyl transferase 1α; DR5, death receptor 5; elF2α, eukaryotic initiation factor 2; ELOVL6, elongase 6; ER, endoplasmatic reticulum; FOXA2, Forkhead Box Protein A2; FABP1, fatty acid binding protein 1; FASN, fatty acid synthase; IRE-1α, inositol-requiring protein-1α; JNK, C-Jun N-terminal kinase; OXPHOS, oxidative phosphorylation; PA, aplmitic acid; PERK, protein kinase RNA-like ER kinase; ROS, reactive oxidative substance; SCD1, stearoyl-CoA desaturase 1; TAG, triacylglceride; XBP1, X-box binding protein-1.
(TIF)

S1 Table Age, BMI, steatosis, inflammation- and fibrosis scores of the cohort studied. Data are shown as median and range of values.
(DOC)

S2 Table Primers for RT-PCR. (DOC)

S1 File Supplementary materials and methods. (DOC)

S2 File NC3Rs ARRIVE guidelines checklist. (PDF)

## References

[1] Tiniakos DG, Vos MB, Brunt EM. Nonalcoholic fatty liver disease: pathology and pathogenesis. Annu Rev Pathol. 2010;5:145–71. doi: 10.1146/annurev-pathol-121808-102132 . 20078219 10.1146/annurev-pathol-121808-102132

[2] Sanyal AJ, Brunt EM, Kleiner DE, Kowdley KV, Chalasani N, Lavine JE, et al Endpoints and clinical trial design for nonalcoholic steatohepatitis. Hepatology. 2011;54(1):344–53. doi: 10.1002/hep.24376 ; 21520200 10.1002/hep.24376 PMC4014460

[3] Doycheva I, Watt KD, Alkhouri N. Nonalcoholic fatty liver disease in adolescents and young adults: The next frontier in the epidemic. Hepatology. 2017;65(6):2100–9. Epub 2017/01/20. doi: 10.1002/hep.29068 . 28103626 10.1002/hep.29068

[4] Bedossa P. Pathology of non-alcoholic fatty liver disease. Liver Int. 2017;37 Suppl 1:85–9. Epub 2017/01/05. doi: 10.1111/liv.13301 . 28052629 10.1111/liv.13301

[5] Fabbrini E, Sullivan S, Klein S. Obesity and nonalcoholic fatty liver disease: biochemical, metabolic, and clinical implications. Hepatology. 2010;51(2):679–89. doi: 10.1002/hep.23280 ; 20041406 10.1002/hep.23280 PMC3575093

[6] Gandhi CR. Augmenter of liver regeneration. Fibrogenesis Tissue Repair. 2012;5(1):10 doi: 10.1186/1755-1536-5-10 22776437 10.1186/1755-1536-5-10 PMC3519801

[7] Thasler WE, Schlott T, Thelen P, Hellerbrand C, Bataille F, Lichtenauer M, et al Expression of augmenter of liver regeneration (ALR) in human liver cirrhosis and carcinoma. Histopathology. 2005;47(1):57–66. doi: 10.1111/j.1365-2559.2005.02172.x 15982324 10.1111/j.1365-2559.2005.02172.x

[8] Senkevich TG, White CL, Koonin EV, Moss B. A viral member of the ERV1/ALR protein family participates in a cytoplasmic pathway of disulfide bond formation [In Process Citation]. Proc Natl Acad Sci U S A. 2000;97(22):12068–73. doi: 10.1073/pnas.210397997 11035794 10.1073/pnas.210397997 PMC17295

[9] Daithankar VN, Farrell SR, Thorpe C. Augmenter of liver regeneration: substrate specificity of a flavin-dependent oxidoreductase from the mitochondrial intermembrane space. Biochemistry. 2009;48(22):4828–37. doi: 10.1021/bi900347v 19397338 10.1021/bi900347v PMC2730831

[10] Farrell SR, Thorpe C. Augmenter of liver regeneration: a flavin-dependent sulfhydryl oxidase with cytochrome c reductase activity. Biochemistry. 2005;44(5):1532–41. doi: 10.1021/bi0479555 15683237 10.1021/bi0479555

[11] Bihlmaier K, Mesecke N, Kloeppel C, Herrmann JM. The disulfide relay of the intermembrane space of mitochondria: an oxygen-sensing system? Ann N Y Acad Sci. 2008;1147:293–302. doi: 10.1196/annals.1427.005 19076451 10.1196/annals.1427.005

[12] Fischer M, Horn S, Belkacemi A, Kojer K, Petrungaro C, Habich M, et al Protein import and oxidative folding in the mitochondrial intermembrane space of intact mammalian cells. Mol Biol Cell. 2013;24(14):2160–70. doi: 10.1091/mbc.E12-12-0862 23676665 10.1091/mbc.E12-12-0862 PMC3708723

[13] Thirunavukkarasu C, Wang LF, Harvey SA, Watkins SC, Chaillet JR, Prelich J, et al Augmenter of liver regeneration: an important intracellular survival factor for hepatocytes. J Hepatol. 2008;48(4):578–88. doi: 10.1016/j.jhep.2007.12.010 18272248 10.1016/j.jhep.2007.12.010 PMC2954779

[14] Cao Y, Fu YL, Yu M, Yue PB, Ge CH, Xu WX, et al Human augmenter of liver regeneration is important for hepatoma cell viability and resistance to radiation-induced oxidative stress. Free Radic Biol Med. 2009;47(7):1057–66. doi: 10.1016/j.freeradbiomed.2009.07.017 19616613 10.1016/j.freeradbiomed.2009.07.017

[15] Francavilla A, Pesetti B, Barone M, Morgano A, Bovenga F, Napoli A, et al Transient GFER knockdown in vivo impairs liver regeneration after partial hepatectomy. Int J Biochem Cell Biol. 2014;53:343–51. doi: 10.1016/j.biocel.2014.05.029 24880092 10.1016/j.biocel.2014.05.029

[16] Shi HB, Sun HQ, Shi HL, Ren F, Chen Y, Chen DX, et al Autophagy in anti-apoptotic effect of augmenter of liver regeneration in HepG2 cells. World J Gastroenterol. 2015;21(17):5250–8. doi: 10.3748/wjg.v21.i17.5250 25954098 10.3748/wjg.v21.i17.5250 PMC4419065

[17] Gandhi CR, Chaillet JR, Nalesnik MA, Kumar S, Dangi A, Demetris AJ, et al Liver-specific deletion of augmenter of liver regeneration accelerates development of steatohepatitis and hepatocellular carcinoma in mice. Gastroenterology. 2015;148(2):379–91. doi: 10.1053/j.gastro.2014.10.008 25448926 10.1053/j.gastro.2014.10.008 PMC4802363

[18] Akazawa Y, Cazanave S, Mott JL, Elmi N, Bronk SF, Kohno S, et al Palmitoleate attenuates palmitate-induced Bim and PUMA up-regulation and hepatocyte lipoapoptosis. J Hepatol. 2010;52(4):586–93. doi: 10.1016/j.jhep.2010.01.003 20206402 10.1016/j.jhep.2010.01.003 PMC2847010

[19] Malhi H, Kaufman RJ. Endoplasmic reticulum stress in liver disease. J Hepatol. 2011;54(4):795–809. doi: 10.1016/j.jhep.2010.11.005 ; 21145844 10.1016/j.jhep.2010.11.005 PMC3375108

[20] Mantzaris MD, Tsianos EV, Galaris D. Interruption of triacylglycerol synthesis in the endoplasmic reticulum is the initiating event for saturated fatty acid-induced lipotoxicity in liver cells. FEBS J. 2011;278(3):519–30. doi: 10.1111/j.1742-4658.2010.07972.x 21182590 10.1111/j.1742-4658.2010.07972.x

[21] Cao SS, Kaufman RJ. Targeting endoplasmic reticulum stress in metabolic disease. Expert Opin Ther Targets. 2013;17(4):437–48. Epub 2013/01/18. doi: 10.1517/14728222.2013.756471 . 23324104 10.1517/14728222.2013.756471

[22] Hotamisligil GS. Endoplasmic reticulum stress and the inflammatory basis of metabolic disease. Cell. 2010;140(6):900–17. doi: 10.1016/j.cell.2010.02.034 ; 20303879 10.1016/j.cell.2010.02.034 PMC2887297

[23] Krautbauer S, Eisinger K, Lupke M, Wanninger J, Ruemmele P, Hader Y, et al Manganese superoxide dismutase is reduced in the liver of male but not female humans and rodents with non-alcoholic fatty liver disease. Exp Mol Pathol. 2013;95(3):330–5. doi: 10.1016/j.yexmp.2013.10.003 24161595 10.1016/j.yexmp.2013.10.003

[24] Eisinger K, Liebisch G, Schmitz G, Aslanidis C, Krautbauer S, Buechler C. Lipidomic analysis of serum from high fat diet induced obese mice. Int J Mol Sci. 2014;15(2):2991–3002. doi: 10.3390/ijms15022991 24562328 10.3390/ijms15022991 PMC3958895

[25] Eisinger K, Krautbauer S, Hebel T, Schmitz G, Aslanidis C, Liebisch G, et al Lipidomic analysis of the liver from high-fat diet induced obese mice identifies changes in multiple lipid classes. Exp Mol Pathol. 2014;97(1):37–43. Epub 2014/05/17. doi: 10.1016/j.yexmp.2014.05.002 . 24830603 10.1016/j.yexmp.2014.05.002

[26] Klissenbauer M, Winters S, Heinlein UA, Lisowsky T. Accumulation of the mitochondrial form of the sulphydryl oxidase Erv1p/Alrp during the early stages of spermatogenesis. J Exp Biol. 2002;205(Pt 14):1979–86. 12089204 10.1242/jeb.205.14.1979

[27] Dayoub R, Wagner H, Bataille F, Stoltzing O, Spruss T, Buechler C, et al Liver regeneration associated protein (ALR) exhibits antimetastatic potential in hepatocellular carcinoma. Mol Med. 2011;17(3–4):221–8. doi: 10.2119/molmed.2010.00117 21152698 10.2119/molmed.2010.00117 PMC3060984

[28] Dayoub R, Groitl P, Dobner T, Bosserhoff AK, Schlitt HJ, Weiss TS. Foxa2 (HNF-3beta) regulates expression of hepatotrophic factor ALR in liver cells. Biochem Biophys Res Commun. 2010;395(4):465–70. doi: 10.1016/j.bbrc.2010.04.023 20382118 10.1016/j.bbrc.2010.04.023

[29] Wolfrum C, Asilmaz E, Luca E, Friedman JM, Stoffel M. Foxa2 regulates lipid metabolism and ketogenesis in the liver during fasting and in diabetes. Nature. 2004;432(7020):1027–32. doi: 10.1038/nature03047 . 15616563 10.1038/nature03047

[30] Xu C, Chakravarty K, Kong X, Tuy TT, Arinze IJ, Bone F, et al Several transcription factors are recruited to the glucose-6-phosphatase gene promoter in response to palmitate in rat hepatocytes and H4IIE cells. J Nutr. 2007;137(3):554–9. 17311939 10.1093/jn/137.3.554

[31] Polimeno L, Pesetti B, Annoscia E, Giorgio F, Francavilla R, Lisowsky T, et al Alrp, a survival factor that controls the apoptotic process of regenerating liver after partial hepatectomy in rats. Free Radic Res. 2011;45(5):534–49. doi: 10.3109/10715762.2011.555482 21291353 10.3109/10715762.2011.555482

[32] Li Y, Wei K, Lu C, Li Y, Li M, Xing G, et al Identification of hepatopoietin dimerization, its interacting regions and alternative splicing of its transcription. Eur J Biochem. 2002;269(16):3888–93. 12180965 10.1046/j.1432-1033.2002.03054.x

[33] Banci L, Bertini I, Cefaro C, Ciofi-Baffoni S, Gajda K, Felli IC, et al An intrinsically disordered domain has a dual function coupled to compartment-dependent redox control. J Mol Biol. 2013;425(3):594–608. doi: 10.1016/j.jmb.2012.11.032 23207295 10.1016/j.jmb.2012.11.032

[34] Gatzidou E, Kouraklis G, Theocharis S. Insights on augmenter of liver regeneration cloning and function. World J Gastroenterol. 2006;12(31):4951–8. doi: 10.3748/wjg.v12.i31.4951 16937489 10.3748/wjg.v12.i31.4951 PMC4087396

[35] Tanigawa K, Sakaida I, Masuhara M, Hagiya M, Okita K. Augmenter of liver regeneration (ALR) may promote liver regeneration by reducing natural killer (NK) cell activity in human liver diseases. J Gastroenterol. 2000;35(2):112–9. 10680666 10.1007/s005350050023

[36] Li Y, Li M, Xing G, Hu Z, Wang Q, Dong C, et al Stimulation of the mitogen-activated protein kinase cascade and tyrosine phosphorylation of the epidermal growth factor receptor by hepatopoietin. J Biol Chem. 2000;275(48):37443–7. doi: 10.1074/jbc.M004373200 10982794 10.1074/jbc.M004373200

[37] Ilowski M, Putz C, Weiss TS, Brand S, Jauch KW, Hengstler JG, et al Augmenter of liver regeneration causes different kinetics of ERK1/2 and Akt/PKB phosphorylation than EGF and induces hepatocyte proliferation in an EGF receptor independent and liver specific manner. Biochem Biophys Res Commun. 2010;394(4):915–20. doi: 10.1016/j.bbrc.2010.03.074 20230786 10.1016/j.bbrc.2010.03.074

[38] Liao XH, Chen GT, Li Y, Zhang L, Liu Q, Sun H, et al Augmenter of liver regeneration attenuates tubular cell apoptosis in acute kidney injury in rats: the possible mechanisms. Ren Fail. 2012;34(5):590–9. doi: 10.3109/0886022X.2012.664470 22417144 10.3109/0886022X.2012.664470

[39] Maehara Y, Fernandez-Checa JC. Augmenter of liver regeneration links mitochondrial function to steatohepatitis and hepatocellular carcinoma. Gastroenterology. 2015;148(2):285–8. doi: 10.1053/j.gastro.2014.12.013 25529802 10.1053/j.gastro.2014.12.013

[40] Ibrahim SH, Kohli R, Gores GJ. Mechanisms of lipotoxicity in NAFLD and clinical implications. J Pediatr Gastroenterol Nutr. 2011;53(2):131–40. doi: 10.1097/MPG.0b013e31822578db 21629127 10.1097/MPG.0b013e31822578db PMC3145329

[41] Thumser AE, Moore JB, Plant NJ. Fatty acid binding proteins: tissue-specific functions in health and disease. Curr Opin Clin Nutr Metab Care. 2014;17(2):124–9. doi: 10.1097/MCO.0000000000000031 . 24500438 10.1097/MCO.0000000000000031

[42] Bechmann LP, Hannivoort RA, Gerken G, Hotamisligil GS, Trauner M, Canbay A. The interaction of hepatic lipid and glucose metabolism in liver diseases. J Hepatol. 2012;56(4):952–64. doi: 10.1016/j.jhep.2011.08.025 . 22173168 10.1016/j.jhep.2011.08.025

[43] Jun DW, Cho WK, Jun JH, Kwon HJ, Jang KS, Kim HJ, et al Prevention of free fatty acid-induced hepatic lipotoxicity by carnitine via reversal of mitochondrial dysfunction. Liver Int. 2011;31(9):1315–24. doi: 10.1111/j.1478-3231.2011.02602.x . 22093454 10.1111/j.1478-3231.2011.02602.x

[44] Guzman C, Benet M, Pisonero-Vaquero S, Moya M, Garcia-Mediavilla MV, Martinez-Chantar ML, et al The human liver fatty acid binding protein (FABP1) gene is activated by FOXA1 and PPARalpha; and repressed by C/EBPalpha: Implications in FABP1 down-regulation in nonalcoholic fatty liver disease. Biochim Biophys Acta. 2013;1831(4):803–18. doi: 10.1016/j.bbalip.2012.12.014 . 23318274 10.1016/j.bbalip.2012.12.014

[45] Matsuzaka T, Atsumi A, Matsumori R, Nie T, Shinozaki H, Suzuki-Kemuriyama N, et al Elovl6 promotes nonalcoholic steatohepatitis. Hepatology. 2012;56(6):2199–208. doi: 10.1002/hep.25932 . 22753171 10.1002/hep.25932

[46] Serviddio G, Bellanti F, Vendemiale G. Free radical biology for medicine: learning from nonalcoholic fatty liver disease. Free Radic Biol Med. 2013;65:952–68. doi: 10.1016/j.freeradbiomed.2013.08.174 . 23994574 10.1016/j.freeradbiomed.2013.08.174

[47] Yamada K, Mizukoshi E, Sunagozaka H, Arai K, Yamashita T, Takeshita Y, et al Characteristics of hepatic fatty acid compositions in patients with nonalcoholic steatohepatitis. Liver Int. 2015;35(2):582–90. doi: 10.1111/liv.12685 . 25219574 10.1111/liv.12685

[48] Lu C, Li Y, Zhao Y, Xing G, Tang F, Wang Q, et al Intracrine hepatopoietin potentiates AP-1 activity through JAB1 independent of MAPK pathway. FASEB J. 2002;16(1):90–2. doi: 10.1096/fj.01-0506fje 11709497 10.1096/fj.01-0506fje

[49] Li Y, Lu C, Xing G, Zhu Y, He F. Macrophage migration inhibitory factor directly interacts with hepatopoietin and regulates the proliferation of hepatoma cell. Exp Cell Res. 2004;300(2):379–87. doi: 10.1016/j.yexcr.2004.07.019 15475002 10.1016/j.yexcr.2004.07.019

[50] Li Y, Liu W, Xing G, Tian C, Zhu Y, He F. Direct association of hepatopoietin with thioredoxin constitutes a redox signal transduction in activation of AP-1/NF-kappaB. Cell Signal. 2005;17(8):985–96. doi: 10.1016/j.cellsig.2004.11.016 15894171 10.1016/j.cellsig.2004.11.016

[51] Dayoub R, Vogel A, Schuett J, Lupke M, Spieker SM, Kettern N, et al Nrf2 activates augmenter of liver regeneration (ALR) via antioxidant response element and links oxidative stress to liver regeneration. Mol Med. 2013;19:237–44. doi: 10.2119/molmed.2013.00027 23887691 10.2119/molmed.2013.00027 PMC3769531

