# Supplementary figures and images for: Attenuated lipotoxicity and apoptosis is linked to exogenous and endogenous augmenter of liver regeneration by different pathways
Source: PLoS One. 2017 Sep 6;12(9):e0184282. doi: 10.1371/journal.pone.0184282 (PMC5587239; doi:10.1371/journal.pone.0184282)

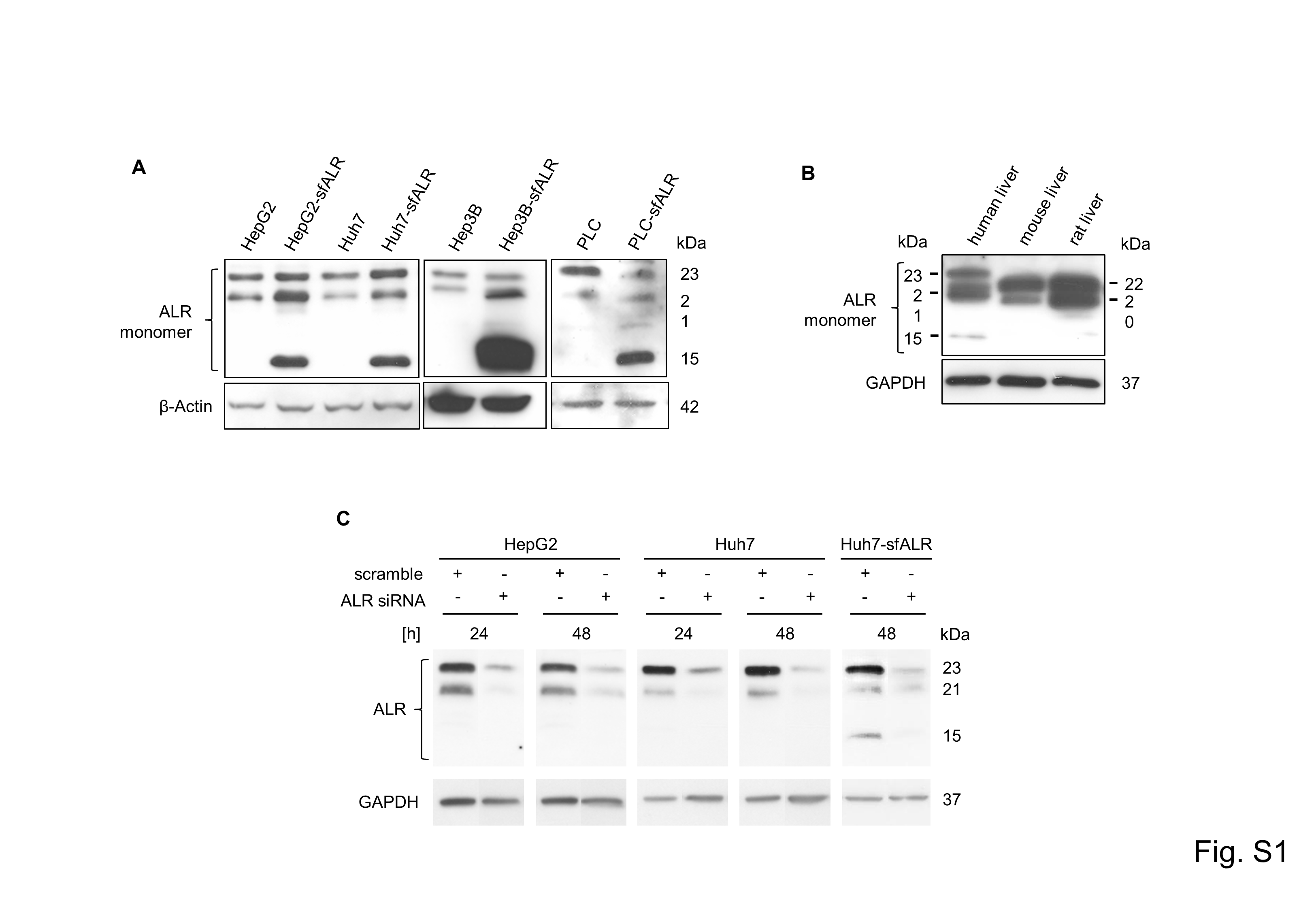

Supplement: S1 Fig — (A) Hepatoma cell lines were transfected with empty vector (pcDNA3.1) or expression plasmid for short form ALR (sfALR, 15 kDa). Positive cell clones stably expressing sfALR (besides 21 and 23 kDa ALR), were selected using G-418 for 2 weeks. (B) Liver tissue homogenates (20 μg) from different species were analyzed for ALR expression performing western blot. Human liver tissue reveals strong bands of 23 and 21 kDa and a weak staining at 15 kDa. Mouse and rat liver show a double band at slightly lower molecular sizes of 22 and 20 kDa, with an additional very faint 15 kDa band in rat liver tissue. (C) HepG2, Huh-7 and Huh-7-sfALR cells were maintained in culture and treated with ALR—siRNA (100 nM) for indicated time points. After 24 and 48 hours ALR expression was analyzed by western blot and demonstrated strong reduction of all ALR isoforms in HepG2, Huh-7 and Huh-7-sfALR cells (one out of three experiments). (TIF) [file pone.0184282.s001.tif]

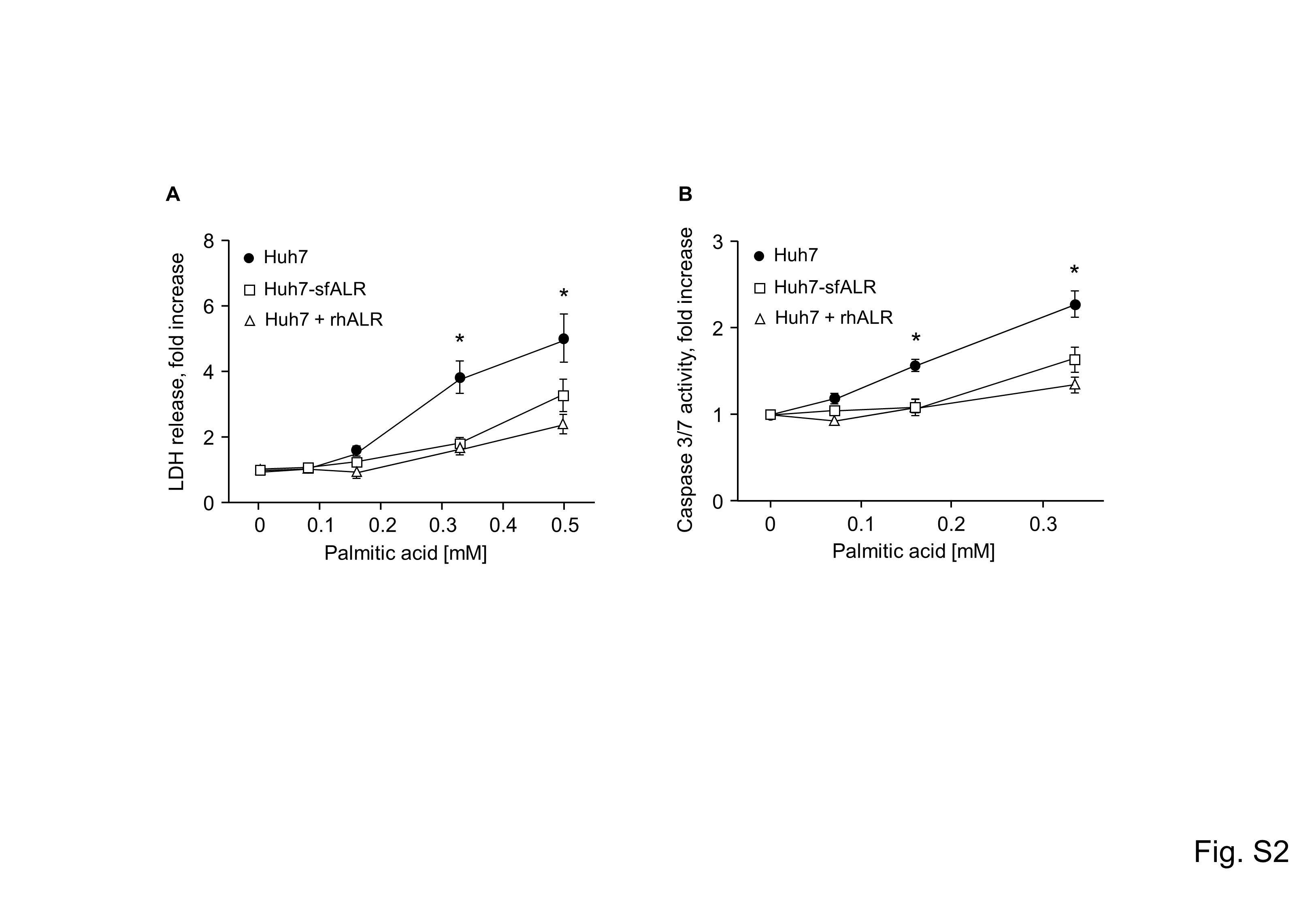

Supplement: S2 Fig — Cultured cells were incubated for 24 hours with indicated concentrations of palmitic acid or vehicle. (A) Lipotoxicity, analyzed by LDH release into supernatant, is reduced in Huh7-sfALR (0.33 mM, 0.5 mM) and Huh7 (0.33 mM, 0.5 mM) pretreated with 100 nM rhALR compared to Huh7 without rhALR treatment. (B) Lipo-apoptosis, analyzed by cellular caspase 3/7 activity, is reduced in Huh7-sfALR (0.16 mM, 0.33 mM) and Huh7 (0.16 mM, 0.33 mM) pretreated with 100 nM rhALR compared to Huh7 without rhALR treatment. (n = 3; * p < 0.05 differs from Huh7-sfALR and Huh7 + rhALR). (TIF) [file pone.0184282.s002.tif]

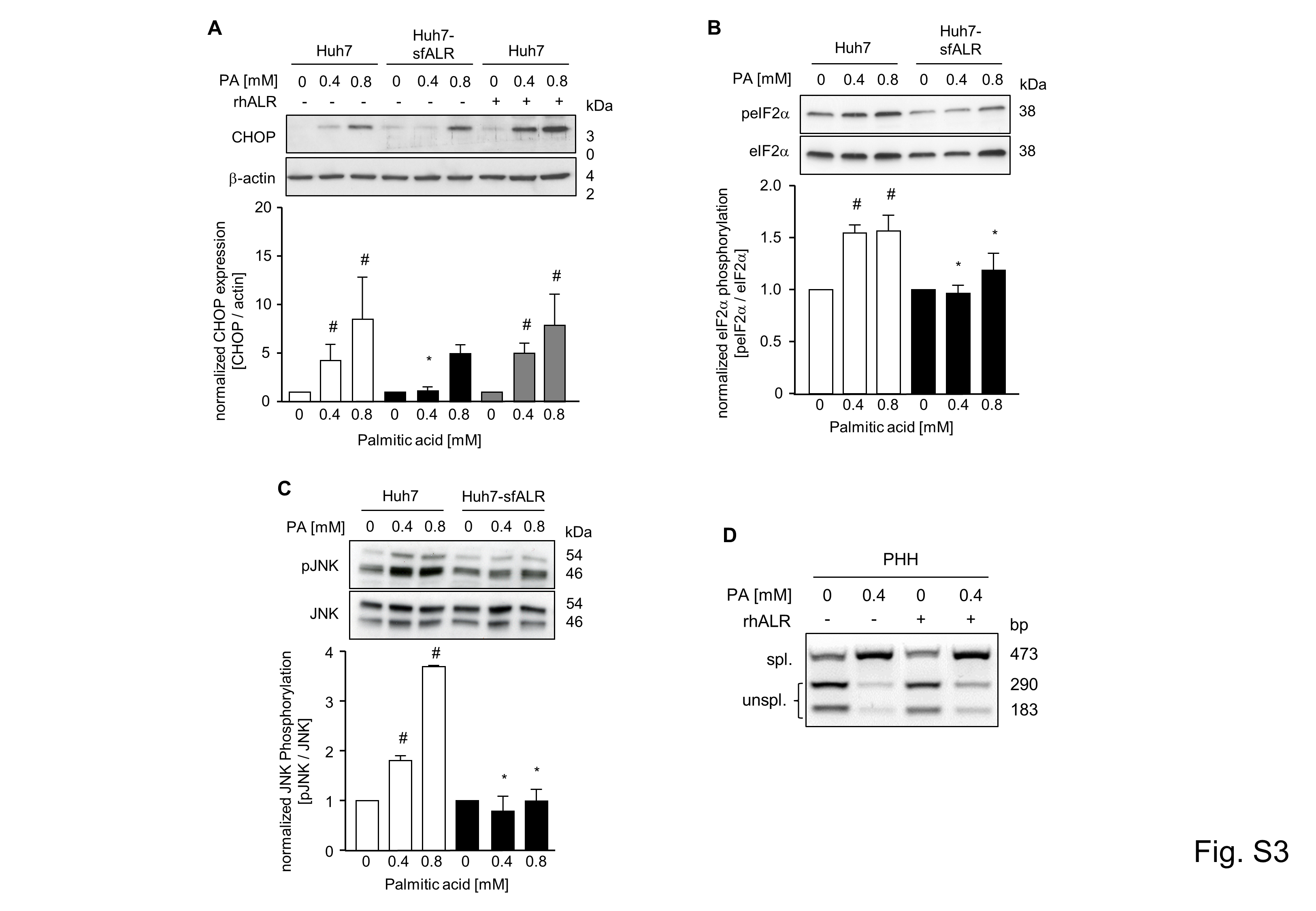

Supplement: S3 Fig — Cultured cells were incubated for 8 hours with indicated concentrations of palmitic acid or vehicle. (A) Expression of CHOP was analyzed by western blotting of Huh7-sfALR and Huh7 cells pretreated without or with 100 nM rhALR followed by densitometric analysis. (B) Activation of elF2α by phosphorylation was analyzed by western blotting of Huh7 and Huh7-sfALR cells followed by densitometric analysis. (n = 3; * p < 0.05 differs from corresponding Huh7, # p < 0.05 differs from 0 mM PA). (C) Activation of JNK by phosphorylation was analyzed by western blotting of Huh7 and Huh7-sfALR cells followed by densitometric analysis. (n = 3; * p < 0.05 differs from corresponding Huh7, # p < 0.05 differs from 0 mM PA). (D) Activation of XBP1 in PHH treated without or with 100 nM rhALR was analyzed by amplifying XBP1 cDNA by PCR following incubation with PstI. Spliced form of XBP1 indicating activation results in a 473 bp amplification product, whereas unspliced form of XBP1 shows digested 290 bp and 183 bp amplification products (one out of three experiments is shown). (TIF) [file pone.0184282.s003.tif]

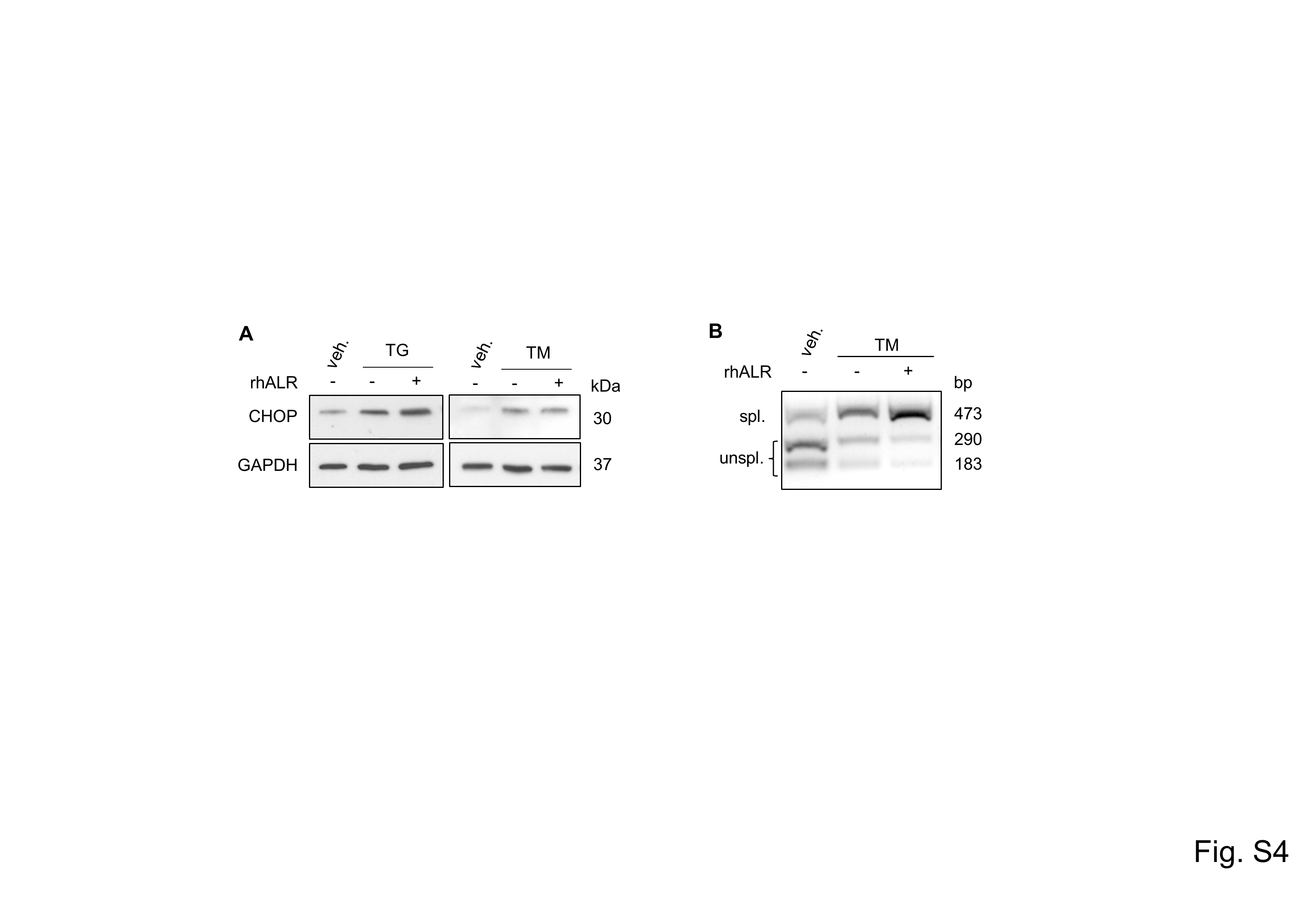

Supplement: S4 Fig — PHH were incubated either for 6 hours with 0.5 μM Thapsigargin or for 16 hours with 10 μg/ml Tunicamycin. (A) Activation of CHOP was analyzed by western blotting of PHH pretreated without or with 100 nM rhALR. (B) Activation of XBP1 in PHH treated without or with 100 nM rhALR was analyzed by amplifying XBP1 cDNA by PCR following incubation with PstI. Spliced form indicating activation results in a 473 bp amplification product, whereas unspliced form of XBP1 shows digested 290 bp and 183 bp amplification products (one out of three experiments is shown). (TIF) [file pone.0184282.s004.tif]

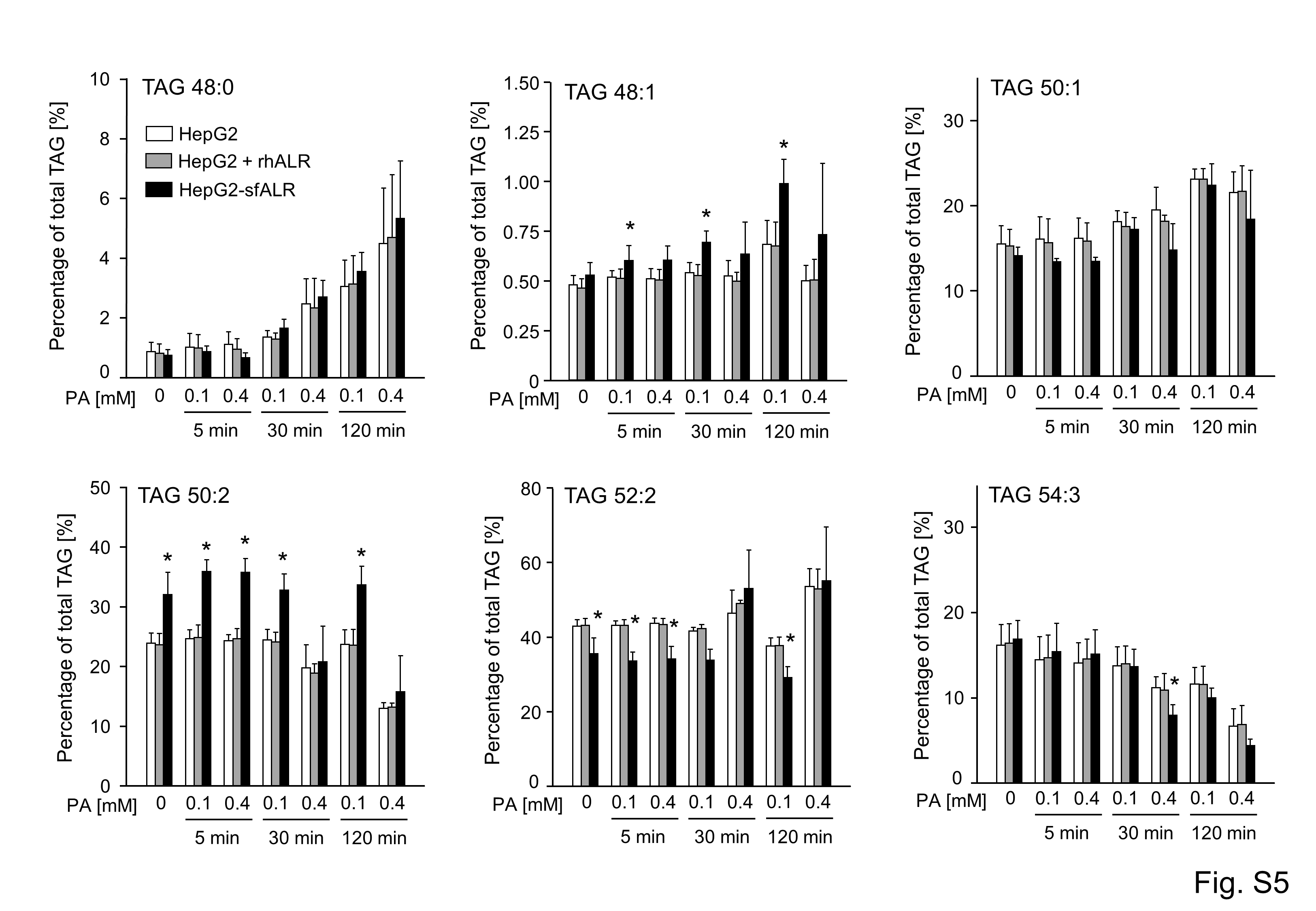

Supplement: S5 Fig — Cultured cells were incubated for 24 hours with indicated concentrations of palmitic acid or vehicle. The most abundant individual TAG (48:0, 48:1, 50:1, 50:2, 52:2 and 54:3) levels were analyzed in HepG2-sfALR, HepG2 cells treated without or with 100 nM rhALR. Data were normalized to HepG2 cells treated with 0 mM PA (n = 3; * p < 0.05 differs from HepG2). (TIF) [file pone.0184282.s005.tif]

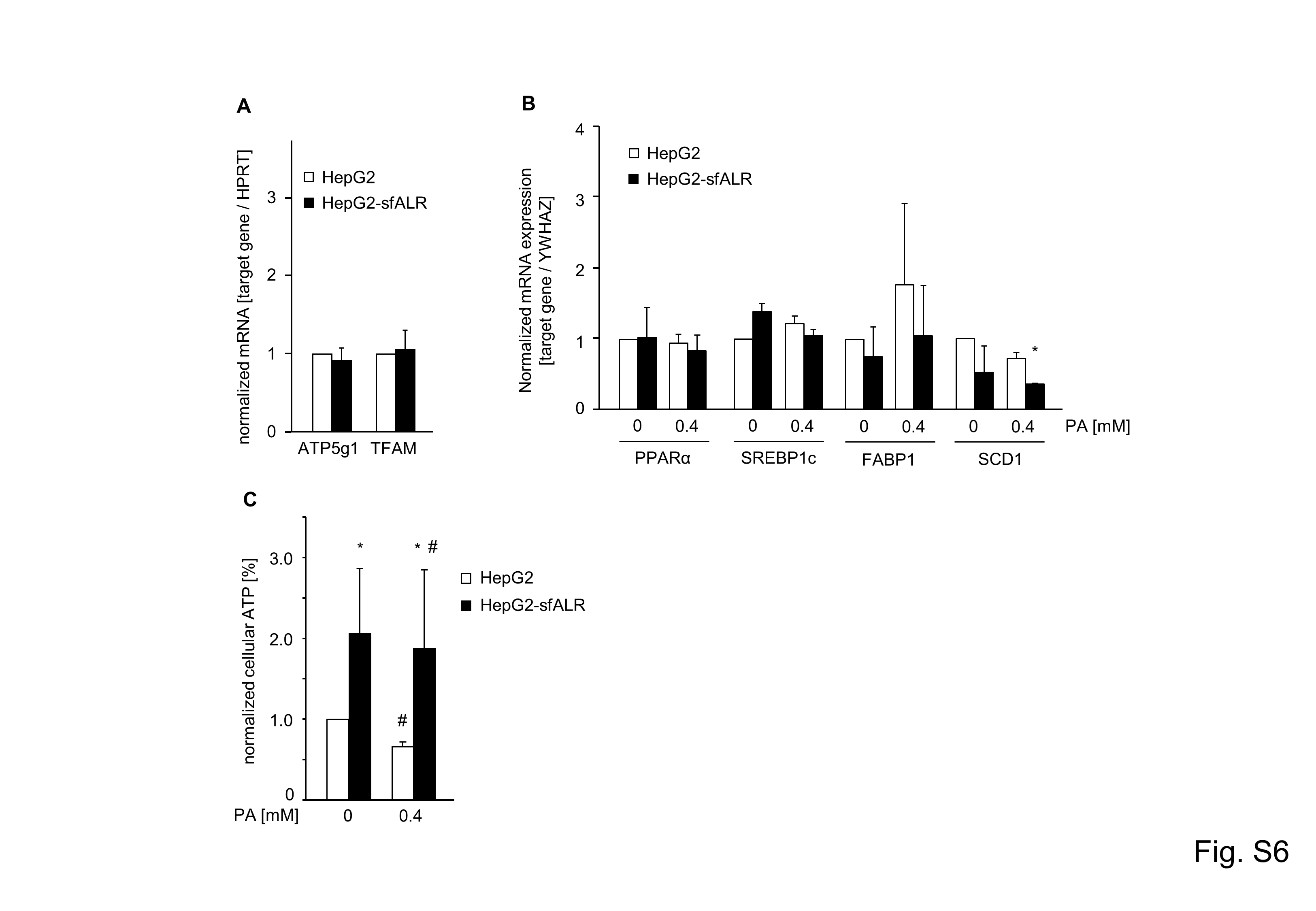

Supplement: S6 Fig — Cultured cells were incubated for 24 hours with indicated concentrations of palmitic acid or vehicle. (A) HepG2-sfALR and HepG2 cells were analyzed for ATP5g1 and TFAM mRNA expression performing qRT-PCR. Atp5g1 encodes a subunit of mitochondrial ATP synthase and TFAM (mTFA) encodes mitochondrial transcription factor A, a key activator of mitochondrial transcription and genome replication. HPRT mRNA expression was determined for normalization (n = 3). (B) HepG2-sfALR and HepG2 cells were incubated for 24 hours with palmitic acid or vehicle and analyzed for lipid metabolism related gene expression (PPARα, SREBP1c, FABP1, SCD1 mRNA) performing qRT-PCR. YWHAZ mRNA expression was determined for normalization. (n = 3; * p < 0.05 differs from HepG2; # p < 0.05 differs from 0 mM PA). (C) Cellular ATP content was determined in Huh7 and Huh7-sfALR cells treated for 24 hours with palmitic acid or vehicle (n = 3; * p < 0.05 differs from Huh7; # p < 0.05 differs from corresponding 0 mM PA). ATP content is almost twice as high in sfALR expressing cells as in control cells. (TIF) [file pone.0184282.s006.tif]

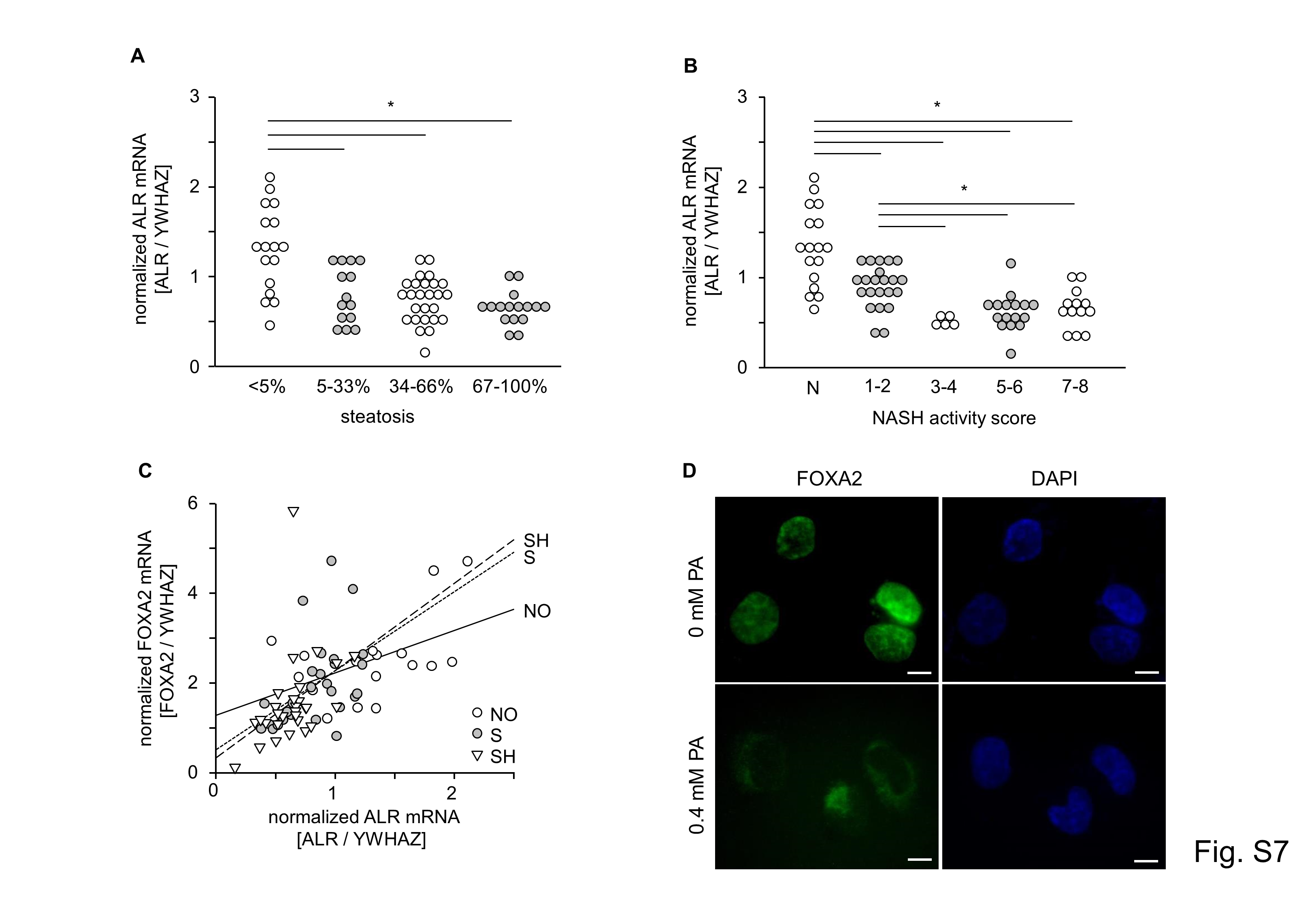

Supplement: S7 Fig — Expression of ALR mRNA was analyzed by qRT-PCR in liver tissue samples from patients with hepatic steatosis or NASH and is plotted in correlation to (A) steatosis grade (<5% = 1.31 ± 0.48; 5–33% = 0.80 ± 0.32; 34–66% = 0.74 ± 0.26; 67–100% = 0.65 ± 0.19) and (B) NASH activity score (normal, N = 1.31 ± 0.48; 1–2 = 0.90 ± 0.24; 3–4 = 0.52 ± 0.06; 5–6 = 0.62 ± 0.21; 7–8 = 0.66 ± 0.23; * p < 0,05 differs significantly. (C) A correlation plot of ALR and FOXA2/HNF3β mRNA expression is shown with best fit lines (Spearman correlation) for NASH (SH, dash line, r2 = 0.168), hepatic steatosis (S, dotted line, r2 = 0.223) and normal (NO, straight line, r2 = 0.235) liver tissue. YWHAZ mRNA expression was determined for normalization. (D) Huh7 cells were incubated for 24 hours with vehicle or 0.4 mM PA and FOXA2/HNF3β translocation from nucleus into cytosol was visualized by immunostaining of FOXA2/HNF3β. (one out of three experiments is shown; bar = 10 μm). (TIF) [file pone.0184282.s007.tif]

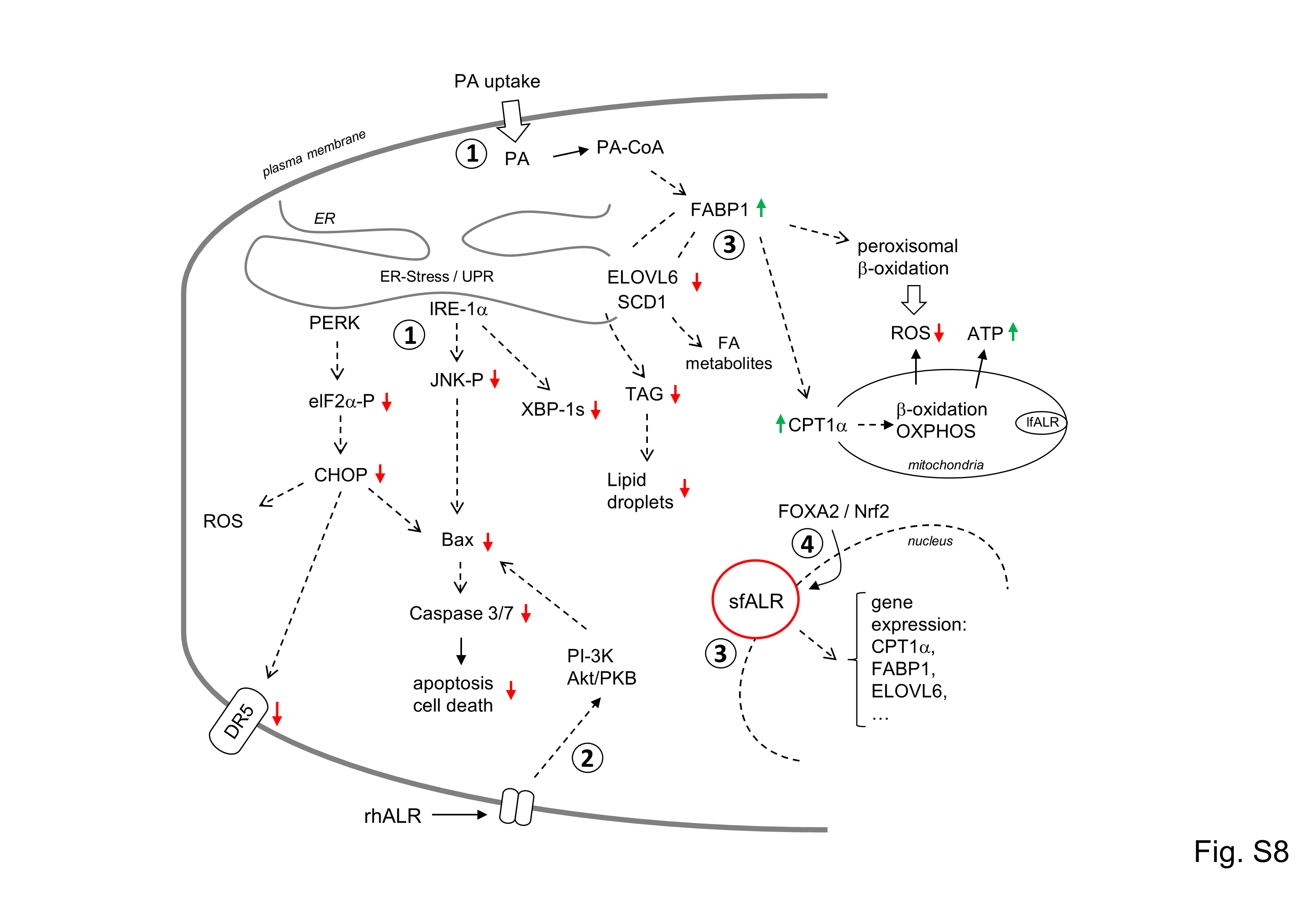

Supplement: S8 Fig — 1) FFA such as PA are taken up by the cell and subsequently converted into fatty acyl-CoA. Fatty acyl-CoA (e.g. PA-CoA) are activated forms of FA that can be either oxidized in mitochondria or utilized in the ER as substrates for the synthesis of phospholipids, cholesterol esters, and TAGs. High levels of PA-CoA results in oversaturated lipid intermediates and TAGs, which is responsible for ER-stress and apoptosis [19, 42]. 2) Exogenous applied recombinant human ALR (rhALR) binds to its receptor leading to PI-3K (Akt/PKB) activation [37], which was shown to be responsible for lower expression of pro-apoptotic bax protein and therefore reduced apoptosis. Exogenously applied rhALR could diminish PA induced lipoapoptosis, but had no impact on PA induced activation of ER-stress response pathways. 3) Expression of endogenous ALR leads to altered expression of genes regulating lipid metabolism and subsequently to reduced ER-stress and lipoapoptosis. Cells expressing sfALR show high expression of FABP1 facilitating substrates for both mitochondria and peroxisomes, but enhanced CPT1α expression might favor mitochondrial β-oxidation accompanied by increased ATP levels. Increased mitochondrial β-oxidation attenuates oxidative stress by reducing alternative oxidation pathways in peroxisomes and microsomes, which results in reduced release of ROS. FABP1 also delivers FA-CoAs to the ER for further TAG synthesis, but increased β-oxidation could abate the supply of PA-CoA and therefore decreases TAG synthesis and lipid droplet size. In addition ALR lowers ELOVL6 expression and therefore less ELOVL6 mediated metabolites, which are responsible for hepatic lipotoxicity [45]. Reduced levels of PA, as a consequence of sfALR expression, result in lower TAG synthesis and in less ER-stress shown by reduced activation of ER membrane sensors of the unfolded protein response such as IRE-1α and PERK, and consecutively CHOP and Bax expression. 4) ALR expression is regulated by its transcriptio [file pone.0184282.s008.tif]
