# Supplementary material for: Attenuated lipotoxicity and apoptosis is linked to exogenous and endogenous augmenter of liver regeneration by different pathways
Source: PLoS One. 2017 Sep 6;12(9):e0184282. doi: 10.1371/journal.pone.0184282 (PMC5587239; doi:10.1371/journal.pone.0184282)
Supplement: S1 Table — Data are shown as median and range of values. (DOC) [file pone.0184282.s009.doc]

**S1** **Table. Age, BMI, steatosis, inflammation- and fibrosis scores of the cohort studied. Data are shown as median and range of values.**

|  | Control | Steatosis | NASH |
| --- | --- | --- | --- |
| Patients (male/female) | 17 (8/9) | 27 (12/15) | 29 (17/12) |
| Age | 60 (20-72) | 59 (35-84) | 64 (33-82) |
| BMI (kg/m^2^) | 24 (20-30) | 29 (24-43) | 29 (21-58) |
| Steatosis | 0 | 2 (1-2) | 3 (1-3) |
| Inflammation | 0 | 0 (0-1) | 2 (1-3) |
| Fibrosis | 0 | 0 (0-3) | 2 (1-3) |
|  |  |  |  |

Human liver tissues for mRNA expression analysis were histologically examined for patients without NAFLD, patients with simple liver steatosis and patients with NASH as described earlier [19]. Briefly, Steatosis, inflammation and fibrosis were histologically examined. Steatosis was scored as <5% steatosis (0), 5 to 33% steatosis (1), >33 to 66% steatosis (2) and >66% steatosis (3). Inflammation was scored as no foci / 20 × field (0), <2 foci / 20 × field (1), 2–4 foci /20×field (2) and >4 foci / 20 × field (3). Fibrosis was defined as no fibrosis (0), zone 3 perisinusoidal/pericellular fibrosis; focally or extensively present (1), zone 3 perisinusoidal/ pericellular fibrosis with focal or extensive periportal fibrosis (2), zone 3 perisinusoidal/pericellular fibrosis and portal fibrosis with focal or extensive bridging fibrosis (3) and cirrhosis (4). The score of each feature was summed up and ranged from 0 to 9. Patients with a score of 5 or above were designed as suffering from NASH.
