# Supplementary material for: Attenuated lipotoxicity and apoptosis is linked to exogenous and endogenous augmenter of liver regeneration by different pathways
Source: PLoS One. 2017 Sep 6;12(9):e0184282. doi: 10.1371/journal.pone.0184282 (PMC5587239; doi:10.1371/journal.pone.0184282)
Supplement: S2 Table — (DOC) [file pone.0184282.s010.doc]

**S2 Table. Primers for RT-PCR.**

| Gene | Accession Nr. | Primer sequence (5’ - 3’) | |
| --- | --- | --- | --- |
| Human ACC | NM_198836 | *Fwd.* | gct gct cgg atc act agt gaa |
|  |  | *Rev.* | ttc tgc tat cag tct gtc cag |
| Human ALR | NM_005262 | *Fwd.* | gaa gcg gga cac caa gtt ta |
|  |  | *Rev.* | ttc agc aca ctc ctc aca gg |
| Mouse ALR | NM_023040 | *Fwd.* | cac agg atc ggg aag aat tg |
|  |  | *Rev.* | att cct cgc agg ggt aaa ac |
| Human ATP5G1 | NM_005175 | *Fwd.* | cat tga cac agc agc caa gt |
|  |  | *Rev.* | gcc aag aat ggc ata gga ga |
| Human CPT1α | NM_001876 | *Fwd.* | ctc agt ggg agc gga tgt tta |
|  |  | *Rev.* | tcg atg gta cac gac gat gtg |
| Human ELOVL6 | NM_024090 | *Fwd.* | gaa gat cag ccc caa tga a |
|  |  | *Rev.* | cag aga cca gag cac taa t |
| Human FABP1 | NM_001443 | *Fwd.* | tgt cgg aaa tcg tgc ag |
|  |  | *Rev.* | gat tat gtc gcc gtt gag tt |
| Human FASN | NM_004104 | *Fwd.* | cag gca cac acg atg gac |
|  |  | *Rev.* | cgg agt gaa tct ggg ttg at |
| Human FOXA2 | NM_021784 | *Fwd.* | ccg ttc tcc atc aac aac ct |
|  |  | *Rev.* | ggg gta gtg cat cac ctg tt |
| Human HPRT | NM_000194 | *Fwd.* | tga cac tgg caa aac aat gca |
|  |  | *Rev.* | ggt cct ttt cac cag caa gct |
| Human PPARα | NM_001001928 | *Fwd.* | ctg gaa gct ttg gct tta cg |
|  |  | *Rev.* | ggg gac cac agg ata agt ca |
| Human SCD1 | NM_005063 | *Fwd.* | cgc tag act tgt ctg acc tag aa |
|  |  | *Rev.* | caa gcg tgg gca gga tga agc a |
| Human SREBP1c | NM_001005291 | *Fwd.* | acg gca gcc cct gta acg acc act gtg a |
|  |  | *Rev.* | tgc caa gat ggt tcc gcc act cac cag g |
| Human TFAM | NM_003201 | *Fwd.* | ccc aga tgc aaa aac tac aga act aa |
|  |  | *Rev.* | tcc gcc cta taa gca tct tga |
| Human YWHAZ | NM_003406 | *Fwd.* | gca att act gag aga caa ctt gac a |
|  |  | *Rev.* | tgg aag gcc ggt taa ttt t |
| Mouse YWHAZ | NM_011740 | *Fwd.* | cgc taa taa tgc agt tac tga gag a |
|  |  | *Rev.* | ttg gaa ggc cgg tta att tt |

Abbreviations: ACC, acetyl-CoA carboxalyse; ALR, augmenter of liver regeneration; ATP5G1, ATP synthase H+ transporting mitochondrial Fo complex subunit C1; CPT1α, carnitine palmitoyl transferase 1α; ELOVL6, elongase 6; FABP1, fatty acid binding protein 1; FASN, fatty acid synthase; FOXA2, Forkhead Box Protein A2; HPRT, hypoxanthine guanine phosphoribosyl transferase; PPARα, peroxisome proliferator-activated receptor α; SCD1, stearoyl-CoA desaturase 1; SREBP1c, sterol response binding protein-1c; TFAM, mitochondrial transcription factor A; YWHAZ, tyrosine 3-monooxygenase/tryptophan 5-monooxygenase activation protein zeta.
