# Supplementary material for: Attenuated lipotoxicity and apoptosis is linked to exogenous and endogenous augmenter of liver regeneration by different pathways
Source: PLoS One. 2017 Sep 6;12(9):e0184282. doi: 10.1371/journal.pone.0184282 (PMC5587239; doi:10.1371/journal.pone.0184282)
Supplement: S1 File — (DOC) [file pone.0184282.s011.doc]

**S1 File. Supplementary Materials and Methods:**

*Cell culture*

HepG2 cells (ATCC® HB-8065™), PLC cells (ATCC® HB-8065™) and Hep3B cells (ATCC® HB-8064™) were obtained from American Type Culture Collection (ATCC) (Manassas, VA), whereas Huh-7 cells (ECACC 01042712) were obtained from  European Collection of Authenticated Cell Cultures (ECACC) (Salisbury, UK ). Human hepatoma cell lines were cultivated as described [1] and stably sfALR expressing cells were prepared as reported earlier [2]. HepG2 and Huh7 cells (5×10^4^ / cm^2^) were seeded in 6-well plates for 24 hours, and after additional 24 h of starvation (serum free culture medium) cells were treated with indicated concentrations of palmitic acid (Sigma-Aldrich, St. Louis, MO) [3] with or without 100 nM rhALR and harvested at indicated time points.

Human liver tissue for cell isolation was obtained from liver resections of patients undergoing partial hepatectomy for metastatic liver tumors of colorectal cancer. Primary human hepatocytes (PHH) were isolated as described earlier [4, 5] and treatment was performed in serum-free medium.

*Antibodies*

Antibodies were available from: Mouse anti-CVα (**# 439800) from Invitrogen (Darmstadt, Germany),** mouse anti-Bax (**#** ABIN306548) from Antikörper-online (Aachen, Germany), mouse anti-HSP70 (# ADI-SPA-810) from Enzo life sciences (Lörrach, Germany), Goat anti-FOXA2 (# sc-6554x), goat anti-FABP1 (# sc-16064) and Donkey anti Goat IgG-FITC (# sc-2024) from Santa Cruz Biotechnology (Heidelberg, Germany), mouse anti-PDI (# ab5484) and rabbit anti-DR5 (# ab8416) from Abcam (Cambridge, UK), rabbit anti-CHOP (# 5554), rabbit anti-JNK (# 9252S), rabbit anti-phospho-JNK (Thr183/Tyr185) (# 9251S), rabbit anti-eIF2-α (# 9722S), rabbit anti-phospho-eIF2-α (# [9721](http://www.cellsignal.com/products/9721.html)S), rabbit anti-SCD1 (# 2438), rabbit anti-CPT1a (# 12252), rabbit anti-GAPDH (# 5174) and rabbit anti-β-actin (# 4970) all from [Cell Signaling Technology](http://www.jbc.org/cgi/redirect-inline?ad=Cell Signaling Technology) (Beverly, MA). Mouse anti-Golgin 97 (# A21270) and MitoTracker® Deep Red FM (# M22426) were from life technologies (Darmstadt, *Germany).* Rabbit anti-ALR was prepared as reported earlier (8)*.* Goat anti mouse IgG-HRP (P0447) and Goat anti rabbit IgG-HRP (P0448) were from Dako (Hamburg ,Germany).

*Analysis of XBP1 splicing*

XBP1 was amplified by PCR using a forward primer (5’-AAACAGAGTAGCAGCTCA GACTGC-3’) and a reverse primer (5’-TCCTTCTGGGTAGACCTCTGGGAG-3’). The PCR product was digested using P*stI* restriction enzyme for 1 hour at 37°C, followed by separation on a 2.5 % agarose gel. The gels were photographed under UV transillumination [3].

*Si-RNA transfection*

For siRNA transfections, HepG2, Huh7 and Huh7-sfALR were grown in 12-well plates (0.2x10^6^ cells per well) and transfected with 100 pmol of siRNA in the presence of Lipofectamine RNAimax reagent (Life Technologies, Darmstadt, Germany), according to the procedure provided by the manufacturer. The siRNA obtained from Life Technologies were, for ALR (NM_005262) the Silencer® Select Pre-designed siRNA (catalog# 4392420, s5702, 5’-UUGCGGUUCACUUCAUUGUgc-3’) and for Silencer® Select Negative Control No. 1 siRNA (catalog# 4390843) which has been used as negative control for sequence independent effect. Afterwards, cells were harvested at indicated time points and prepared for further analysis.

*Triacylglyceride content and palmitic acid uptake*

Cells were treated with either palmitic acid or [13C] labelled palmitic acid (UL-13C16, Larodan Fine Chemicals AB, Sweden) as described before. Cell extraction for Triacylglyceride and [13C] palmitic acid analysis was performed as described elsewhere [6]. Briefly, after FFA treatment cells were placed on ice and washed twice with ice-cold PBS and water, each, followed by scraping off the plate. From the suspension in the residual water 50 µl was removed for cell quantification by DNA determination and 200 µl was mixed with 500 µl methanol containing 1 mM EDTA followed by storage at -80°C until analysis.

After thawing, the mixture was spiked with internal standard and extracted with chloroform as described previously [7]. The chloroform extract was evaporated to dryness and reconstituted in 2-propanol. LC-MS analysis of TAGs was performed on a 6550 iFunnel QTOF mass spectrometer (Agilent Technologies, Waldbronn, Germany) equipped with a Dual Agilent Jet Stream (AJS) electrospray source. Electrospray parameters were as follows: gas temperature, 200 °C; drying gas flow, 11 l/min; nebulizer pressure, 35 psig; sheath gas temp, 350 °C; sheath gas flow, 12 l/min; capillary voltage, 4000 V; nozzle voltage, 500 V; and fragmentor voltage, 350 V. Mass spectrometric analysis was done in positive ion mode with a scan range of m/z 100–1100 and a scan rate of 4 spectra/s. Data was acquired with intensity thresholds of 10 counts/0.001 % and stored in profile mode. Chromatographic separation of the triglycerides was carried out at 70°C on a Poroshell 120 RP18 column (2.1 × 100 mm, 2.7 μm particle size, Agilent) coupled to a 1290 Infinity UHPLC System (Agilent). Gradient runs of mobile phase A (20 mM ammonium acetate in acetonitrile:water 95：5 (v/v)) and mobile phase B (20 mM ammonium acetate in 2-propanol) were programmed as follows: 0-2 min, 5-15 % B; 2-4 min, 15 % B; 4-8 min, 15-50 % B; 8-11 min, 50-60 % B; 11-12 min, 60 % B, 12-13 min, 60-90 % B ;13-21 min, 90 % B. The column was reconditioned to initial conditions (5% B) for 5 min. TAGs were analyzed as ammonium adducts [M+NH4]^+^ using triheptadecanoin as internal standard. Calibration samples were prepared in aqueous BSA in the concentration range from 0.0625 to 75 nmol/10 µl and were worked up as described above, and analyzed together with the unknown samples. Calibration curves based on internal standard calibration were obtained by weighted (1/x) quadratic regression for the peak-area ratio of the analyte to the internal standard against the amount of the analyte. The concentration of the analytes in unknown samples was obtained from the regression curve. Assay accuracy and precision were determined by analyzing quality controls that were prepared like the calibration samples.

Total uptake of [13C] labelled palmitic acid was determined after acid hydrolysis of the lipid extract. An aliquot of the extract in 2-propanol was diluted with an equal volume of water and treated with a mixture of acetonitrile and concentrated hydrochloric acid (4:1 v/v) for 2 h at 90 °C. The fatty acids were extracted with hexane, the hexane extract was evaporated to dryness and reconstituted in mobile phase. LC-MS-MS analysis of [13C] labelled myristic acid, palmitic acid, palmitoleic acid, stearic acid and oleic acid was carried out similar to a described method [8] using heptadecanoic acid as internal standard.

*Statistical analysis*

All data are presented as mean values +/- standard deviation. Statistical analysis for non parametric data was done by two-tailed Mann-Whitney U Test, Student’s t-test for paired samples, and value of p < 0.05 was regarded as significant (SPSS Statistics 21.0 program, IBM, Leibiz Rechenzentrum, München, Germany) The Spearman correlation was calculated using the IBM SPSS statistics 21.0 program.

**References**

1. Dayoub R, Vogel A, Schuett J, Lupke M, Spieker SM, Kettern N, et al. Nrf2 activates augmenter of liver regeneration (ALR) via antioxidant response element and links oxidative stress to liver regeneration. Mol Med. 2013;19:237-44. doi: molmed.2013.00027 [pii];10.2119/molmed.2013.00027 [doi].

2. Dayoub R, Wagner H, Bataille F, Stoltzing O, Spruss T, Buechler C, et al. Liver regeneration associated protein (ALR) exhibits antimetastatic potential in hepatocellular carcinoma. Mol Med. 2011;17(3-4):221-8.

3. Akazawa Y, Cazanave S, Mott JL, Elmi N, Bronk SF, Kohno S, et al. Palmitoleate attenuates palmitate-induced Bim and PUMA up-regulation and hepatocyte lipoapoptosis. J Hepatol. 2010;52(4):586-93. doi: S0168-8278(10)00011-5 [pii];10.1016/j.jhep.2010.01.003 [doi].

4. Damm G, Pfeiffer E, Burkhardt B, Vermehren J, Nussler AK, Weiss TS. Human parenchymal and non-parenchymal liver cell isolation, culture and characterization. Hepatol Int. 2013;7:951-8.

5. Weiss TS, Dayoub R. Thy-1 (CD90)-Positive Hepatic Progenitor Cells, Hepatoctyes, and Non-parenchymal Liver Cells Isolated from Human Livers. Methods Mol Biol. 2017;1506:75-89.

6. Haynes CA, Allegood JC, Sims K, Wang EW, Sullards MC, Merrill AH, Jr. Quantitation of fatty acyl-coenzyme As in mammalian cells by liquid chromatography-electrospray ionization tandem mass spectrometry. J Lipid Res. 2008;49(5):1113-25. doi: D800001-JLR200 [pii];10.1194/jlr.D800001-JLR200 [doi].

7. Wu H, Southam AD, Hines A, Viant MR. High-throughput tissue extraction protocol for NMR- and MS-based metabolomics. Anal Biochem. 2008;372(2):204-12.

8. Twardowski L, Cheng F, Michaelsen J, Winter S, Hofmann U, Schaeffeler E, et al. Enzymatically Modified Low-Density Lipoprotein Is Present in All Stages of Aortic Valve Sclerosis: Implications for Pathogenesis of the Disease. J Am Heart Assoc. 2015;4(10):e002156.
